# P11 deficiency increases stress reactivity along with HPA axis and autonomic hyperresponsiveness

**DOI:** 10.1038/s41380-020-00887-0

**Published:** 2020-10-01

**Authors:** Vasco C. Sousa, Ioannis Mantas, Nikolas Stroth, Torben Hager, Marcela Pereira, Haitang Jiang, Sandra Jabre, Wojciech Paslawski, Oliver Stiedl, Per Svenningsson

**Affiliations:** 1 Department of Clinical Neuroscience, Karolinska Institutet, Stockholm, Sweden; 2 Department of Functional Genomics, Center for Neurogenomics and Cognitive Research, Amsterdam Neuroscience, VU University, Amsterdam, The Netherlands; 3 Department of Psychosomatics and Psychiatry, Zhongda Hospital, Medical School of Southeast University, Nanjing, PR China; 4 Department of Molecular and Cellular Neurobiology, Center for Neurogenomics and Cognitive Research, Amsterdam Neuroscience, VU University, Amsterdam, The Netherlands

**Keywords:** Neuroscience, Depression

## Abstract

Patients suffering from mood disorders and anxiety commonly exhibit hypothalamic–pituitary–adrenocortical (HPA) axis and autonomic hyperresponsiveness. A wealth of data using preclinical animal models and human patient samples indicate that p11 deficiency is implicated in depression-like phenotypes. In the present study, we used p11-deficient (p11KO) mice to study potential roles of p11 in stress responsiveness. We measured stress response using behavioral, endocrine, and physiological readouts across early postnatal and adult life. Our data show that p11KO pups respond more strongly to maternal separation than wild-type pups, even though their mothers show no deficits in maternal behavior. Adult p11KO mice display hyperactivity of the HPA axis, which is paralleled by depression- and anxiety-like behaviors. p11 was found to be highly enriched in vasopressinergic cells of the paraventricular nucleus and regulates HPA hyperactivity in a V_1B_ receptor-dependent manner. Moreover, p11KO mice display sympathetic–adrenal–medullary (SAM) axis hyperactivity, with elevated adrenal norepinephrine and epinephrine levels. Using conditional p11KO mice, we demonstrate that this SAM hyperactivity is partially regulated by the loss of p11 in serotonergic neurons of the raphe nuclei. Telemetric electrocardiogram measurements show delayed heart rate recovery in p11KO mice in response to novelty exposure and during expression of fear following auditory trace fear conditioning. Furthermore, p11KO mice have elevated basal heart rate in fear conditioning tests indicating increased autonomic responsiveness. This set of experiments provide strong and versatile evidence that p11 deficiency leads to HPA and SAM axes hyperresponsiveness along with increased stress reactivity.

## Introduction

A healthy neuroendocrine and autonomic response to stress, regulated by the hypothalamic–pituitary–adrenocortical (HPA) and the sympathetic–adrenal–medullary (SAM) axes, respectively, are essential functions that evolved as a mechanism for surviving and coping with acute physical stress [1, 2]. Sustained stress, on the other hand, is a strong environmental risk factor for developing major depression, due to the adverse effects from prolonged exposure to high levels of adrenal steroid hormones as well as epinephrine (adrenaline) and norepinephrine (noradrenaline) [3–5]. Patients suffering from generalized anxiety, major depression, and other mood disorders commonly exhibit HPA axis hyperactivity, manifested as high concentrations of corticotropin-releasing hormone (CRH) and exacerbated response to adrenocorticotropic hormone (ACTH) [6]. Clinical studies indicate that patients with anxiety and depressive disorders have increased risk for cardiovascular disease [7]. A reduction in beat-to-beat heart rate (HR) variability has been linked with post-traumatic stress disorder [8], which is associated with exaggerated SAM responsiveness to stressful conditions [9].

P11 (also named S100A10) is a member of the S100 family [10, 11] and has an important role in the pathophysiology of depression [12]. Mice with p11 deficiency, via genetic deletion or RNAi knockdown, display a depressive-like phenotype [12, 13]. P11 is known to work as a multitarget adapter protein that regulates intracellular proteins and increases the surface expression of several transmembrane effector proteins, such as serotonin receptors (5HTR) 1B, 1D and 4, mGluR5, and various ion channels [14]. Several studies have shown that deletion of p11 in brain areas that express these receptors induces depression- and anxiety-like behavioral traits and/or reduces therapeutic responsiveness to antidepressants [15–19]. Moreover, there is evidence that p11 expression is dysregulated in chronically stressed animals in a circuitry-dependent manner with a downregulation of p11 in prefrontal cortex, but an upregulation in the lateral habenula [20, 21]. There are no studies that have investigated the role of p11 in the regulation of the HPA and SAM axes, despite their critical roles in the development of depressive disorders. To assess the importance of p11 expression to HPA and SAM axes functions, we measured the response of p11-deficient mice to various stressors using behavioral, endocrine, and physiological readouts across early postnatal and adult life.

## Methods and materials

### Animals

Wild-type (WT), constitutive p11 knockout (p11KO) and conditional knockout of p11 in serotonin transporter (SERT)-expressing neurons (SERT-p11cKO) were generated as previously described [12, 22] on a C57BL/6J background, and bred as detailed in the Supplementary Information (SI). There was no genotype effect on body weight in pups or adult mice. All experiments were approved by the Karolinska Institutet Ethical Committee and the VU University Amsterdam according to Swedish and Netherlands guidelines, respectively, in full compliance with European requirements.

### Pup ultrasonic vocalizations (USVs)

Littermate mice from homozygous breeding pairs were subjected to unpredictable maternal separation combined with unpredictable maternal stress for 3 h daily, from postnatal day (PND) 1–14 (MS) [23], or were left undisturbed (control). USVs were recorded using a condenser microphone connected to the Avisoft Ultra Sound Gate 116Hb system (Avisoft Bioacoustics, Glienicke, Germany) over a period of 5 min from pups of both sexes, on PNDs 3, 6, 9, and 12. See SI for a detailed description.

### Adult behavioral phenotyping

Behavioral tests were performed in non-maternally separated 10-week-old mice to assess depressive- and anxiety-like phenotypes (detailed in SI).

### Drug administration

The corticotropin-releasing hormone receptor 1 (CRH_1_) antagonist CP 154,526 (30 mg/kg), or arginine vasopressin (AVP) receptor 1B (V_1B_) antagonist TASP 0390325 (3 mg/kg) (Tocris Bioscience, Bristol, UK), dissolved in sterile saline solution containing 5% TWEEN 80 (vehicle), were administered i.p. (10 ml/kg) 30 min before mice were subjected to the forced swim test. The dosages were based on the original characterization of these ligands [24, 25], and following pilot studies to test for inhibition of stress-induced ACTH.

### Response to acute swim stress, serum collection, and quantification of hormones

Mice were placed into water tanks (temperature 24 ± 1 °C) for 7-min before euthanization by decapitation immediately (within 1 min) or following a 24-min rest. Trunk blood serum was collected for ACTH and corticosterone enzyme-linked immunosorbent assays. See SI for a detailed description.

### RNA extraction, reverse transcription, and quantitative PCR (qPCR)

Hypothalamic tissue samples were collected as described in the SI, then subsequently disrupted in lysis buffer (RNeasy Mini kit, Qiagen, Hilden, Germany) using an ultrasonic processor (EpiShear Probe Sonicator, Active Motif, La Hulpe, Belgium). Total RNA was subsequently extracted, processed, and quantified as detailed in the SI.

### Fluorescent and radioactive in situ hybridization

Fresh frozen, post-fixed brain sections were hybridized with ^35^S-radiolabeled antisense riboprobes against p11, and activity-regulated cytoskeleton protein (ARC) genes; or RNAscope^®^ ZZ probes (Advanced Cell Diagnostics, Abingdon, Oxford) against p11, AVP, cortocotropin releasing hormone (CRH), AVP receptor 1b (V_1B_), ARC, 5-hydroxytryptamine receptor 1b (5-HT_1B_), tyrosine hydroxylase (Th), tryptophan hydroxylase 2 (Tph2), and counterstained with DAPI (Advanced Cell Diagnostics), or immunostained with a polyclonal ChAT primary antibody (catalog# AB144P, Millipore, Solna, Sweden), and an anti-goat Alexa Fluor 568 secondary antibody (Invitrogen, Stockholm, Sweden). Described in detail in the SI.

### High-performance liquid chromatography

Catecholamine content of mouse adrenal glands was measured by high-performance liquid chromatography (HPLC). See SI for full details.

### Radio-telemetry, novelty exposure, and auditory trace fear conditioning

Adult male mice were implanted with an ECG radio-transmitter (Data Sciences International, St. Paul, MN, USA) and subjected to novelty exposure and to auditory trace fear conditioning with a 30-s trace interval and a 2-s shock of 0.7 mA (constant current) to determine HR responses indicative of unconditioned and conditioned fear, respectively [26]. See SI for detailed protocol.

### Experimental design and Statistical analysis

The sample sizes were based on previous reports to ensure adequate power. The experimenter was blinded to group allocation during experiment, and analysis was automated. Animals were pseudo-randomly allocated to experimental groups, so that each cage would contain balanced group distribution. Outliers in the data were calculated using the Grubb’s test calculator tool from GraphPad (GraphPad, San Diego, CA, USA), and removed. Normality of the data distribution was checked with a Normal probability plot (InVivoStat program [27]). Statistical analysis was carried out by two-way or three-way analysis of variance (ANOVA) or repeated measures ANOVA followed by Fisher’s least significance difference (LSD) post-test, where indicated, using the InVivoStat program [27]. All data were plotted using GraphPad Prism 8 and presented as mean ± SEM of the number of subjects/samples per group as detailed in the Supplementary Table 1.

## Results

### Separation-induced USVs are exacerbated in p11KO pups

**Fig. 1:**
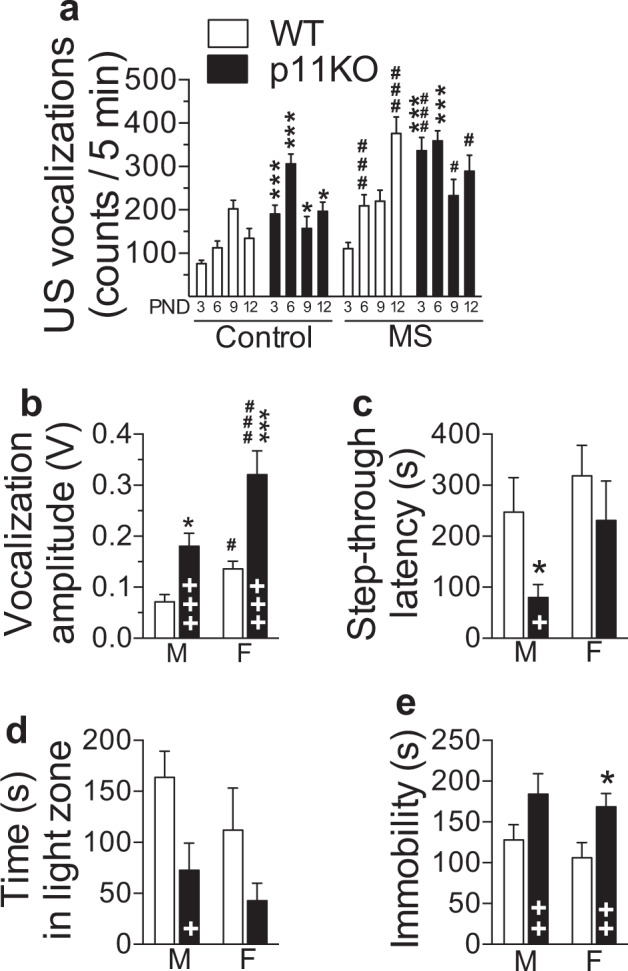
Increased anxiety- and depression-like phenotype of p11 KO mice. **a** P11KO pups display increased ultrasonic vocalizations (USVs). The number of USVs over a period of 5 min was recorded at PND3, 6, 9, and 12 in wild-type (WT) and p11 knockout (p11KO) pups (mixed sex, see Supplementary Information). Significant effects of genotype (*p* < 0.001, *F*_(1,134)_ = 38.38), rearing (*p* < 0.001, *F*_(1,134)_ = 46.5) and age (*p* = 0.0001, *F*_(3,392)_ = 7.14) as well as genotype × age (*p* < 0.001, *F*_(3,392)_ = 23.99), rearing × age (*p* < 0.01, *F*_(3,392)_ = 4.92) and genoty*p*e × rearing × age (*p* < 0.01, *F*_(3,392)_ = 5.33) interaction. All data represent mean ± SEM. *p < 0.05, ***p < 0.001, versus corresponding WT; ^#^*p* < 0.05, ^###^*p* < 0.001, rearing difference within genotype; calculated by three-way ANOVA followed by Fisher’s LSD test. PND postnatal day. **b** Sound intensity of vocalizations was measured over 5 s immediately post-shock in the training trial of the passive avoidance (PA) test. P11KO mice vocalized more intensely (louder) after a mild electric shock exposure during PA training. Significant effects of genotype (*p* < 0.0001, *F*_(1,29)_ = 21.75) and sex (*p* < 0.0001, *F*_(1,29)_ = 20.10). **c** Step-through latency in the passive avoidance retention test 24 h following training was lower in p11KO mice (*p* < 0.05, *F*_**(**1,34)_ = 6.17). **d** Time spent in the light compartment of the 5-min light–dark exploration test indicated an anxiety-like phenotype in p11KO mice of both sexes (genotype effect, *p* < 0.05, *F*_(1,32)_ = 6.83). **e** Cumulative immobility time (floating) in the forced swim test confirmed a depressive-like phenotype in p11KO mice (*p* < 0.01, *F*_(1,31)_ = 9.28). All data represent mean ± SEM. ^+^*p* < 0.05, ^++^*p* < 0.01, ^+++^*p* < 0.001 overall genotype difference; *p < 0.05, ***p < 0.001, versus corresponding WT; ^#^*p* < 0.05, ^###^*p* < 0.001, sex difference within genoty*p*e; calculated by two-way ANOVA, followed by Fisher’s LSD test.

The number of separation-induced pup USVs was measured for 5 min at PND 3, 6, 9, and 12 from litters undergoing daily maternal separation (MS) and from non-stressed (control) litters. Under both rearing conditions, p11KO pups emitted a larger number of USVs compared to WT pups (*p* < 0.001, Fig. 1a). MS increased the number of USVs in both genotypes (*p* < 0.001). Although, as expected [28], the overall number of USVs varies with age (*p* = 0.0001), peak vocalization occurred significantly earlier in p11KO than in WT pups (*p* < 0.001). In both genotypes, MS caused higher USVs at earlier postnatal stages (*p* < 0.01). Finally, our data show an age-dependent gene × environment interaction (*p* < 0.01). In accordance with the well-known stress hyporesponsive period [29], ACTH levels did not reflect the pups USVs (Supplementary Fig. 1a), which suggests that the USVs are a behavioral response differentially regulated from HPA axis activity in this stage of development. We also found that p11KO dams largely display similar maternal care as WT dams, except for enhanced nest building in p11KO dams (Supplementary Fig. 1b–e).

### Anxiety- and depression-related behaviors are enhanced in adult p11KO mice

The reaction to a mild shock, applied as an unconditioned stimulus during the passive avoidance training session, was recorded and analyzed for vocalization of mice based on sound amplitude (Fig. 1b). In both sexes, p11KO mice emitted louder vocalizations than corresponding WT mice (*p* < 0.0001_,_ Fig. 1b). In both genotypes, overall vocalization amplitude was higher in females than males (*p* < 0.0001, Fig. 1b). Despite reacting more strongly to the electric shock during training (Fig. 1b), p11KO mice displayed an overall shorter step-through latency 24 h post-training (*p* < 0.05, Fig. 1c). Male p11KO mice displayed 68% shorter step-through latencies than WT mice (*p* < 0.05, Fig. 1c). The magnitude of this effect is comparable to that previously reported [16]. Female p11KO mice also tended to display shorter step-through latencies compared to WT counterparts (Fig. 1c), despite having markedly exacerbated post-shock reactivity (Fig. 1b).

In the light–dark (L/D) box test, p11KO mice of both sexes spent less time in the light compartment (*p* < 0.05, Fig. 1d), indicating an anxiety-like phenotype. In line with previous studies [12, 30], behavioral despair, as measured by immobility in the forced swim test, is also more pronounced in p11KO mice of both sexes (*p* < 0.01, Fig. 1e), despite p11KO mice showing higher locomotor activity in the open field test (Supplementary Fig. 2). Taken together, our results indicate that p11KO mice are more stress-responsive, passive coping [31], anxiety-, and depressive-like than their WT counterparts.

### HPA axis activation is enhanced in p11KO mice due to paraventricular nucleus (PVN) hyperactivity

**Fig. 2:**
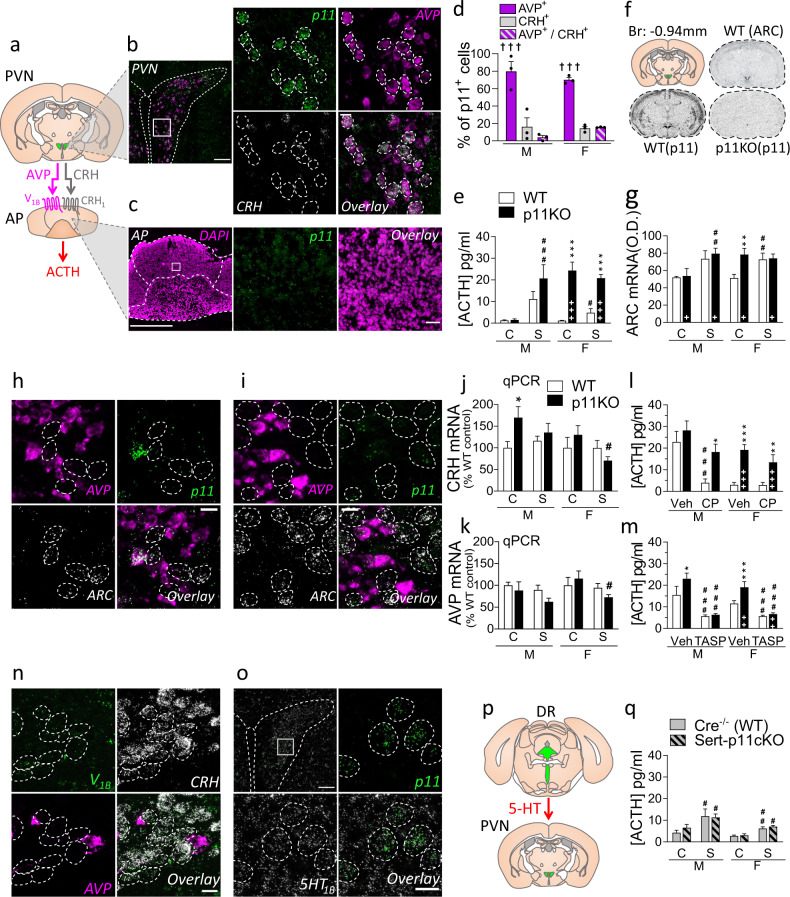
p11 induced changes in the HPA axis. **a** Arginine-vasopressin (AVP) and corticotropin-releasing hormone (CRH) are released from the paraventricular nucleus (PVN) of the hypothalamus, through the hypophyseal portal system into the anterior pituitary (AP), where they activate V_1B_ and CRH_1_ receptors, respectively, to induce adrenocorticotropic hormone (ACTH) release. **b** Fluorescent in situ hybridization (FISH) images in the PVN show that p11 transcripts (green) are strongly expressed in the PVN, and overlap with AVP mRNA (magenta), but not CRH mRNA (white). Scale bars: 100 µm on the left, 15 µm on the right. Dashed line: p11 and AVP co-expressing cells. **c** In the AP, where cell bodies are stained with DAPI (magenta), FISH shows very low p11 expression (green). Scale bars: 500 µm on the left, 30 µm on the right. **d** The percentage of p11 expressing cells (p11^+^) that co-express AVP (AVP^+^) was significantly higher (*p* < 0.001, *F*_(2,12)_ = 62.33) than those co-expressing CRH (CRH^+^), or both AVP and CRH (AVP^+^/CRH^+^). Two-way ANOVA showed no significant sex difference in the co-expression ratios. **e** Serum concentrations of ACTH were measured at baseline (C) or 1 min post-stress (S), in male (M) and female (F) p11KO and corresponding WT mice. In p11KO females, there is a sizable hypersecretion of ACTH (*p* < 0.0001, *F*_(1,33)_ = 116.38) at baseline and 1 min post-stress compared to WT. Male p11KO mice showed higher but not statistically significant post-stress ACTH than WT. **f** Representative in situ autoradiograms at −0.94 mm from bregma with antisense p11 and ARC probes. The diagram in the top left delineates the PVN (green), from which optical density values (corrected for background) were measured. The top right autoradiogram is representative of ARC mRNA, taken from WT mice and illustrates the expression of this gene in the PVN. The bottom autoradiograms show the WT expression of p11 mRNA, and the corresponding section in a p11KO mouse. **g** Quantitative measurements of ARC gene expression at baseline (C) or 15 min post-stress (S) were performed by radioactive in-situ hybridization. There was a significant overall genotype (*p* < 0.05, *F*_(1,31)_ = 4.5) and group (*p* < 0.001, *F*_(1,31)_ = 17.6) difference. FISH images in the PVN of WT (**h**), and p11KO (**i**) unstressed females showing that ARC (white) mRNA is not co-expressed in cells positive for AVP (magenta) and p11 (green). Scale bars: 15 µm. Dashed line: ARC expressing cells. **j** CRH and **k** AVP mRNA abundance in the PVN was measured at baseline (C) or 1 min post-stress (S) by quantitative polymerase chain reaction (qPCR). For CRH mRNA, there was a significant overall sex difference (*p* < 0.05, *F*_(1,58)_ = 4.92) and a genotype × stress interaction (*p* < 0.05, *F*_(1,58)_ = 4.39). For AVP mRNA, there was only a significant overall stress difference (*p* < 0.05, *F*_(1,58)_ = 5.36). Levels of ACTH 1 min post-swim stress, measured 30 min following injection of vehicle (Veh) and **l** CRH_1_ receptor antagonist CP 154,526 (CP, 30 mg/kg, i.p.); or **m** V_1B_ antagonist, TASP 0390325 (TASP, 3 mg/kg, i.p.). **l** In males, CP could reduce ACTH in WT but not in p11KO mice (*p* < 0.0001, *F*_(1,25)_ = 22.51); In females, there was a genotype difference (*p* < 0.0001, *F*_(1,25)_ = 30.44), but CP did not significantly lower ACTH in WT females as Veh-treated levels in this cohort were low. **m** TASP could significantly reduce both male (*p* < 0.0001, *F*_(1,31)_ = 35.82) and female (*p* < 0.0001, *F*_(1,32)_ = 51.10) ACTH in both genotypes. Females also show significant effects of genotype (*p* < 0.01, *F*_(1,32)_ = 11.01) and genotype × treatment (*p* < 0.05, *F*_(1,32)_ = 6.57). **n** FISH images in the PVN show that V_1B_ receptor transcripts (green) are present in CRH positive (white), but not AVP positive (magenta) cells. Scale bar: 15 µm. Dashed line: CRH-expressing cells. **o** FISH from PVN showing that 5-HT_1B_ receptor transcripts (white) are widely expressed in the PVN, where they colocalize with p11 transcripts (green). Scale bars: 100 µm on the left, 15 µm on the right. Dashed line: p11 and 5-HT_1B_ co-expressing cells. **p** The PVN receives serotonergic innervation from the dorsal raphe (DR). **q** Levels of ACTH in SERT-p11cKO were not significantly different from WT. There was only a significant effect of stress (Males: *p* < 0.01, *F*_(1,44)_ = 9.58; Females: *p* < 0.0001, *F*_(1,31)_ = 31.40). All data represent mean ± SEM. ^+^*p* < 0.05, ^++^*p* < 0.01, ^+++^*p* < 0.001 overall genotype difference; ^**†††**^*p* < 0.0001, versus CRH^+^ and AVP^+^/CRH^+^ (**d**); *p < 0.05, **p < 0.01, ***p < 0.001, versus corresponding WT; ^**#**^*p* < 0.05, ^**##**^*p* < 0.01, ^**###**^*p* < 0.001, versus baseline (**e, g, j**–**k**, **q**) or versus vehicle (**l**, **m**) within genotype; calculated with a three-way ANOVA followed by Fisher’s LSD test. Two-way ANOVA were performed for each sex in **e**, **l**, **m**, **q**, given that sexual differences in ACTH are a well-known fact.

The secretion of CRH and AVP from PVN neurons is at the origin of the HPA axis response [4]. CRH and AVP regulate ACTH release from the pituitary through activation of CRH_1_ and V_1B_ receptors, respectively. Using double fluorescent in situ hybridization (FISH), we observed robust expression of p11 in the PVN but very low expression level in anterior pituitary cells (Fig. 2a–c). Our results show that p11 positive neurons are distributed in the medial parvocellular PVN (Fig. 2b), where they are localized in a significantly much higher proportion of AVP expressing neurons (nearly 80%, *p* < 0.001) than in CRH or CRH and AVP-expressing neurons (Fig. 2d).

We compared serum ACTH concentrations at baseline (non-stressed, control) and 1-min post-stress in p11KO mice (Fig. 2e). Female, but not male, p11KO mice had >20-fold higher baseline ACTH compared to WT mice (Fig. 2e). Swim stress induced a rapid elevation of ACTH in males of both genotypes and in female WT mice (Fig. 2e). Baseline ACTH levels in female p11KO mice appear to have reached a ceiling effect that was not surpassed post-stress. Male p11KO mice showed twofold higher post-stress ACTH than WT mice, although not statistically significant (Fig. 2e). Serum corticosterone measurements from the same cohort showed similar differences (Supplementary Fig. 3).

We quantified the expression of ARC, as an indication of excitatory input upon PVN cells [32, 33] (Fig. 2f, g). We found that unstressed (control) p11KO female mice show ~50% higher constitutive ARC mRNA expression in the PVN than the WT counterparts (*p* < 0.01, Fig. 2g). Post-hoc analysis found no genotype differences in ARC mRNA expression at baseline among males (Fig. 2g). Acute swim stress induced a strong activation of ARC mRNA expression in the PVN of both genotypes (*p* < 0.001, Fig. 2g). However, in males, post-hoc analysis shows that only p11KO mice have significantly higher post-stress ARC mRNA expression, when compared to baseline (*p* < 0.01, Fig. 2g). Male WT mice were close (*p* = 0.068) to have induced ARC mRNA expression post-stress. There was a significant increase of post-stress ARC mRNA expression (*p* < 0.01, Fig. 2g) in female WT mice, whereas female p11KO mice may have reached a ceiling effect already in unstressed conditions which was not surpassed post-stress. The latter result is reminiscent of the results found for ACTH and corticosterone levels. Even though p11 is predominately expressed in AVP cells, we observed that out of 84 cells clearly positive for ARC mRNA, 77 were negative for AVP in WT females (Fig. 2h). Likewise, out of 103 ARC mRNA expressing cells, 97 were negative for AVP in p11KO females (Fig. 2i). These results suggest that lack of p11 expression in AVP cells might affect the local intra-PVN somatodendritic AVP release and subsequently enhance the activity of CRH neurons.

We then measured the expression of CRH (Fig. 2j) and AVP (Fig. 2k) mRNA from the PVN using qPCR. Statistical analysis of CRH mRNA expression showed a significant sex difference (*p* < 0.05) and genotype × stress (*p* < 0.05) interaction (Fig. 2j). In unstressed males, there was 70% higher CRH expression in p11KO mice as compared to WT mice (*p* < 0.05, Fig. 2j), which were not affected by stress. In females there was a significant difference between stressed and control p11KO females *(p* < 0.05, Fig. 2j). We found no evidence for differential expression of AVP between genotypes of either sex (Fig. 2k), although there was a significant effect of stress in female p11KO mice (*p* < 0.05, Fig. 2k). Activation of gene transcription is required for compensatory biosynthesis of CRH and AVP peptide, released post-stress [34]. However, it is known that, while CRH transcription precedes that of AVP [35], increases in mRNA levels of CRH and AVP are only visible within hours following acute stress [34–36]. It would require a separate time course experiment to accurately quantify all changes in gene expression of CRH and AVP post-stress.

To further investigate how absence of p11 affects CRH and AVP functions, we injected p11KO mice with the selective CRH_1_ antagonist CP 154,526 (CP, 30 mg/kg, i.p.) 30 min prior to the swim test (Fig. 2l). In line with previous studies [24], CP significantly reduced post-stress ACTH in male WT mice (*p* < 0.001, Fig. 2l). At least partly due to a lower baseline, we could not measure any significant effect of CP on post-stress ACTH levels in female WT mice (Fig. 2l). In both sexes, blockade of CRH_1_ failed to reduce ACTH in p11KO mice (Fig. 2l). In contrast, pretreatment with the selective vasopressin V_1B_ receptor antagonist (TASP, 3 mg/kg, i.p.) significantly reduced ACTH levels in both genotypes and sexes (*p* < 0.001, Fig. 2m).

Using FISH, we confirmed [37, 38] that V_1B_ transcripts are enriched in CRH cells (Fig. 2n). Since V_1B_ receptors stimulates signal transduction and PVN neurons are glutamatergic and excitatory, these data indicate a stimulatory effect of somatodendritic AVP upon CRH cells (Fig. 2n). The observation of a strong V_1B_ expression in the CA2 hippocampal region, as previously reported [39], validates the specificity of the signal (Supplementary Fig. 4). To better understand how p11 may regulate the intra-PVN network, we analyzed the expression of the 5-HT_1B_ serotonin receptor, an important binding partner of p11 [12], in the PVN. We found that 5-HT_1B_ transcripts are widely expressed in the PVN (Fig. 2o), where we found 5-HT_1B_ mRNA expressed in 100% of 300 p11 mRNA positive neurons. These data indicate a possible role of the 5-HT_1B_-p11 complex in the control AVP release and implicates a reduced inhibitory action of 5-HT_1B_ receptors in excitatory AVP cells in p11KO mice.

One of the brain areas with the highest expression of p11 transcripts is the dorsal raphe (DR, Fig. 2p, Supplementary Fig. 5) [12, 40]. To test whether the observed HPA axis dysregulation in the global p11KO mice is regulated by altered p11 function in serotonergic neurons, we studied the acute stress response in mice with conditional knockout of p11 in neurons expressing the SERT (Fig. 2q, Supplementary Fig. 5). However, ACTH levels in SERT-p11cKO mice did not differ significantly from WT mice neither under unstressed nor post-stress conditions (*p* > 0.05, Fig. 2q).

### SAM axis activation is enhanced in p11KO mice

**Fig. 3:**
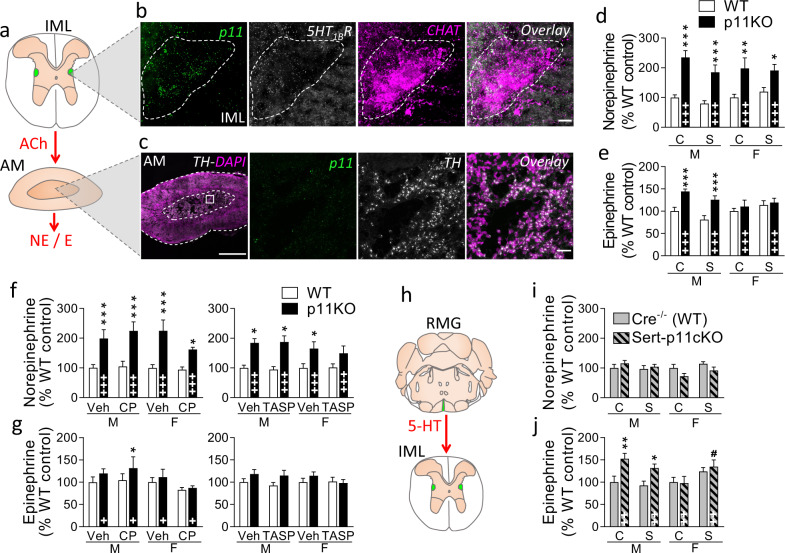
p11 induced changes in the SAM axis. **a** Preganglionic sympathetic neurons from the intermediolateral nucleus (IML) of the thoraco–lumbar spinal cord project to the adrenal medulla (AM), where they release acetylcholine (ACh), which regulates norepinephrine (NE) and epinephrine (E) levels. **b** Fluorescent in situ hybridization (FISH) images of the IML show that p11 transcripts (green) are strongly present in choline acetyltransferase (ChAT) expressing neurons (magenta) and overlap with 5-HT_1B_ mRNA (white). Scale bars: 15 µm. Dashed line: p11, 5-HT_1B_, and ChAT co-expressing neurons. **c** In the AM, where cell bodies are stained with DAPI (magenta), FISH shows no expression of p11 (green) in tyrosine hydroxylase (TH) expressing neurons (white). Scale bars: 500 µm on the left, 30 µm on the right. Adrenal levels of **d** norepinephrine and **e** epinephrine were measured by HPLC at baseline (C) or 1 min post-stress (S), in male (M) and female (F) p11KO and corresponding WT mice. Both catecholamines were more abundant in p11KO compared to WT mice under baseline conditions. There was a significant overall genotype difference in adrenal norepinephrine (**d**) and epinephrine (**e**) (*p* < 0.0001, *F*_(1,62)_ = 42.38 and *p* < 0.001, *F*_(1,62)_ = 14,97, respectively). For epinephrine there was a significant genotype × sex interaction (*p* < 0.05, *F*_(1,62)_ = 6,44). 1 min post-stress levels of adrenal **f** norepinephrine and **g** epinephrine, measured 30 min following injection of vehicle (Veh) and CRH_1_ receptor antagonist CP 154,526 (CP, 30 mg/kg, i.p.); or V_1B_ antagonist, TASP 0390325 (TASP, 3 mg/kg, i.p.). There were no significant differences in norepinephrine or epinephrine levels following either antagonist. There were significantly higher noradrenaline (**f**) levels in p11KO compared to WT mice (*p* < 0.0001, *F*_(1,52)_ = 40.99, left graph; *p* < 0.001, *F*_(1,62)_ = 15.47, right graph). Genotype differences in epinephrine (**g**) levels were statistically significant in one of the cohorts (*p* < 0.05, *F*_(1,51)_ = 6.60, left graph), where there was also a sex difference (*p* < 0.05, *F*_(1,51)_ = 5.78, left graph). **h** Serotonergic neurons from the raphe magnus (RMG), where p11 is highly expressed, project to the IML. Levels of adrenal **i** norepinephrine and **j** epinephrine, at baseline (C) or 1 min post-stress (S), in male (M) and female (F) SERT-p11cKO and corresponding WT mice (Cre^−/−^). In SERT-p11cKO, there were no significant effects for norepinephrine (**i**), while for epinephrine (**j**) there were effects of genotype (*p* < 0.0001, *F*_(1,59)_ = 17.62), genotype × sex (*p* < 0.01, *F*_(1,59)_ = 8.92) and stress × sex interaction (*p* < 0.01, *F*_(1,59)_ = 7.30). Data are shown as the mean ± SEM. ^+^*p* < 0.05, ^++^*p* < 0.01, ^+++^*p* < 0.001 overall genotype difference; *p < 0.05, **p < 0.01, ***p < 0.001, versus corresponding WT; ^**#**^*p* < 0.05, versus baseline; calculated with a three-way ANOVA followed by Fisher’s LSD test.

Having established HPA hormone hypersecretion, we then studied whether p11 deficiency could also affect the SAM system. We assessed the expression of p11 in preganglionic sympathetic neurons and adrenal medulla (Fig. 3a–c). Dense p11 mRNA signal was observed in cholinergic neurons of the intermediolateral nucleus (IML) in the spinal cord (Fig. 3b), while no p11 transcripts were found in the catecholaminergic cells of the adrenal medulla (Fig. 3c). In addition, we observed the presence of 5-HT_1B_ transcripts in IML cells (Fig. 3b).

Measurements of adrenal norepinephrine (Fig. 3d) and epinephrine (Fig. 3e) levels by HPLC show that the global p11KO mice have overall higher levels of both norepinephrine and epinephrine (*p* < 0.0001, Fig. 3d, e). Post-hoc analyses revealed that norepinephrine levels were higher in both female and male p11KO mice (*p* < 0.001, Fig. 3d), while epinephrine was only significantly higher in male p11KO mice (*p* < 0.001, Fig. 3e), where we identified a genotype × sex interaction (*p* < 0.01).

To verify whether these differences in catecholamines stem from the p11-dependent changes we observed in the HPA axis, we analyzed adrenal norepinephrine (Fig. 3f and Supplementary Fig. 6) and epinephrine (Fig. 3g and Supplementary Fig. 6) levels from acutely stressed WT and p11 KO animals treated with the selective CRH_1_ antagonist (CP, 30 mg/kg, i.p.) and the V_1B_ antagonist (TASP, 3 mg/kg, i.p.), 30 min prior to the acute swim stress. For norepinephrine (Fig. 3f), there were no differences in either of the treatments, while higher norepinephrine levels were still observed in p11KO mice (*p* < 0.001). For epinephrine (Fig. 3g), there were also no differences in either of the treatments. A caveat is that the epinephrine levels observed in p11KO mice were not as increased in these cohorts as in the previous cohort presented in Fig. 3e. Nevertheless, these results suggest that p11 deficiency leads to SAM hyperactivity through HPA axis-independent mechanisms.

p11 and 5-HT_1B_ transcripts are robustly expressed throughout the entire raphe nuclei [12] (Supplementary Fig. 5a–c). We therefore examined the impact of p11 deficiency in the raphe magnus (RMG)-IML pathway [41] (illustrated in Fig. 3h), using the SERT-p11cKO mice, to assess whether serotonergic cells expressing p11 can regulate adrenal catecholamines. Results show that norepinephrine levels in SERT-p11cKO mice were not different from WT mice (*p* > 0.05, Fig. 3i and Supplementary Fig. 6). However, in contrast, epinephrine levels were significantly higher in male SERT-p11cKO mice (*p* < 0.001, Fig. 3j and Supplementary Fig. 6), where we also noticed a genotype × sex (*p* < 0.01) interaction, reminiscent of what was observed in the global p11KO mice. Swim stress altered adrenal epinephrine in female SERT-p11cKO mice (*p* < 0.05, Fig. 3j). Taken together, our results suggest that epinephrine levels are affected by p11 deficiency in the RMG-IML pathway, while norepinephrine levels are regulated by p11-dependent mechanisms outside the serotonin system.

### Evidence for hyperresponsiveness of HR regulation in male p11KO mice

**Fig. 4:**
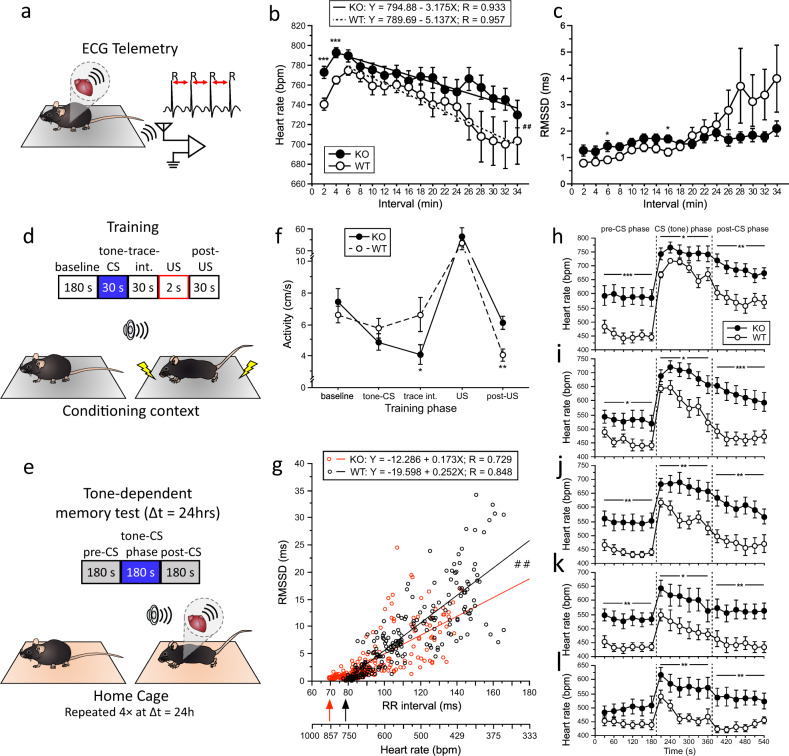
P11KO mice display heightened novelty and fear-conditioned heart rate response. **a** Animation showing the settings of electrocardiographic (ECG) telemetry system. The heart rate (HR) was calculated from male mice by measuring the R–R interval duration. **b** HR and **c** root mean square of the successive differences (RMSSD) (heart rate variability) values during novelty exposure as a function of genotype. Analysis of the genotype × time interaction from 6–34 min indicated a significantly faster drop (^**##**^*p* < 0.01) of HR as shown by the increased negative slope in WT than in p11KO mice. Time effect (*p* < 0.0001, *F*_(1,16)_ = 12.00), genotype effect (*p* = 0.085, *F*_(1,26)_ = 3.21), genotype × time interaction (*p* = 0.074, *F*_(1,16)_ = 1.57). This is supported by the profound increase of HR variability from 26 min onwards (**c**). Genotype effect (*p* = 0.39, *F*_(1,26)_ = 0.76), time effect (*p* < 0.0001, *F*_(1,16)_ = 6.26), genotype × time interaction (*p* < 0.0001, *F*_(1,16)_ = 3.19). Motor activity did not differ between genotypes (data not shown). Animation depicting the auditory trace fear conditioning experimental design, with training (**d**) and retention tests (**e**) that were repeated four times at 24-h interval. **f** Similar training activities were recorded during 180-s baseline, 30-s tone (CS) and 2-s shock (US) exposure, whereas p11KO mice displayed a significantly lower activity (*p < 0.05, *F*_1,27_ = 4.28) than WT mice during the 30-s trace interval and significantly higher activity (**p < 0.002, *F*_1,27_ = 12.19) in the 30-s post-US phase. **g** Linear correlation analysis of the RR interval and the RMSSD value in the tone-dependent retention test (retention test 1) as a function of genotype. Highly significant linear correlations (*p* < 0.0001) were obtained for the slopes of both genotypes which differed significantly (^**##**^*p* < 0.01) between genotypes indicating a lower increase in heart rate variability in p11KO than in WT mice with decreasing HR. Moreover, p11KO mice exhibited a significantly higher maximum heart rate than WT mice (arrows) exceeding the commonly observed maximum range of ~800 bpm in C57BL/6 mice by >50 bpm. Data are shown from the 18 30-s bins of 14 KO mice (252 points) and 13 WT mice (234 points). **h**–**l** HR responses in the tone-dependent retention tests as a function of genotype. Genotype effect (RT1-4: *p* < 0.001, *F*_1,25_ < 15.47; RT5: *p* < 0.005, *F*_1,25_ = 9.66). Both genotypes responded with a significant CS-induced HR increase from baseline values (0–180 s) in retention test 1 (RT1; **h**) to retention test 5 (RT5; **l**). However, the slopes of the HR recovery across CS and post-CS phases were significantly lower (*p* < 0.001 and *p* < 0.005, respectively) in p11KO than in WT mice in RT1 (**h**) and RT2 (**i**). The gradual decrease of the tone-induced HR increase from RT1 to RT5 indicated the extinction of the conditioned auditory fear response. Baseline HR values were significantly higher in p11KO than in WT mice in RT1 to RT4 but not in RT5. Note the reduced HR range shown from **h**–**l**. All data are shown as the mean ± SEM of *n* ≥ 13 males per genotype; *p < 0.05, **p < 0.01, ***p < 0.001.

HR is regulated by both the autonomic nervous system and intrinsic cardiac excitability. HR responses during novelty exposure (Fig. 4a–c) indicated a stronger response to novelty exposure in p11KO mice. Regression analysis indicated a significantly slower HR recovery (*p* = 0.0077) to baseline values in p11KO than in WT mice from min 6–34 of the novelty test (Fig. 4b). HR variability, determined by root mean square of the successive differences (RMSSD) values, did not differ between genotypes, but significantly differed across time (*p* < 0.0001) and showed a genotype × time interaction (*p* < 0.0001; Fig. 4c). The slower HR recovery in p11KO mice was concomitant with a lower increase of HR variability.

Auditory trace fear conditioning experiments (Fig. 4d, e) indicated similar behavioral responses during training (e.g. baseline activity and shock responsiveness) except for decreased activity during the trace interval and increased post-shock activity in p11KO mice (Fig. 4f). The latter may suggest increased panic-like arousal in p11KO mice. During the five retention tests (RT1-5; Fig. 4h–l), p11KO mice exhibited significantly higher HR than WT mice. The p11KO mice responded with higher conditioned stimulus (CS)-induced tachycardia as well as slower HR recovery than WT mice. Higher baseline HR and concomitantly decreased HR variability (RMSSD, data not shown) in p11KO mice was observed during the retention tests 1–4 (Fig. 4h–k) but not retention test 5 (Fig. 4l). Since tone-dependent tests were started around 30 min after the cage transfer, this suggests that the transfer served as a profound unspecific arousing stimulus, leading to a slower recovery of baseline HR than in any study performed by us before with either C57BL/6 mice, genetically modified mice and pharmacological interventions e.g. [26, 42–44].

The correlation of the RR intervals versus RMSSD values (Fig. 4g) obtained in the tone-dependent retention test showed highly significant (WT: *R* = 0.848; p11KO: *R* = 0.729; *p* < 0.0001 for both genotypes) inverse linear relation between HR (RR interval) and its variability (RMSSD) in both genotypes (Fig. 4g) as reported before e.g. [42, 44]. The HR increase is associated with a decrease in HR variability. However, the steepness of slope of the linear regression of the correlation analysis was significantly (*p* < 0.01) lower in p11KO than in WT mice indicating lower HR variability increase in p11KO than in WT mice with decreasing HR values. Furthermore, we propose that this heightened HR may stem both from increased tone of brain SAM centers and intrinsic cardiac excitability as we found a low amount of p11 transcripts in murine cardiac tissue (Supplementary Fig. 7).

## Discussion

This study identifies an HPA axis hyperresponsivity that involves p11 deficiency in AVP expressing neurons and is correlated with increased behavioral stress reactivity. We also provide evidence of autonomic hyperresponsivity in p11 deficiency, where p11 appears to regulate more than one pathway mediating SAM axis activity.

We observed an increased stress reactivity in p11KO mice from the early postnatal period. While all pups displayed low ACTH levels characteristic of the stress hyporesponsive period [29], p11KO pups show an exacerbated response to litter separation, with peak levels developing at an earlier age than in WT counterparts. Furthermore, we identified a gene × environment interaction, whereby p11KO pups subjected to daily maternal separation displayed exacerbated USVs even earlier, and at higher intensity than maternally deprived WT pups. The fact that p11KO mice display such high distress anxiety levels at an early age suggests that p11 regulates the behavioral response to litter separation under the stress hyporesponsive period. Epigenetic alterations with long-term behavioral consequences occur in the early postnatal period [45]. Altered methylation of the p11 promoter with consequences on p11 levels have been reported [46, 47], therefore future studies are needed to better understand the role of p11 in epigenetic regulation of stress vulnerability. The lack of genotype differences in maternal care indicates that the higher anxiety displayed by p11KO pups is indeed driven by endogenous factors and not by poor maternal care. Since maternal behavior is strongly influenced by pup USVs [48, 49], enhanced nest building in p11KO dams may reflect a response to higher pup USVs. Nevertheless, we cannot fully exclude that the strong differences in stress hormones observed in female p11KO mice could, at least partially, render an influence onto offspring during lactation.

The avoidance of the light compartment in the L/D box test shows that p11KO mice have an increased level of anxiety. Furthermore, p11KO mice showed a higher unconditioned response both in trace fear conditioning tests as well as in the passive avoidance. Despite this, they displayed an impaired conditioned response in the passive avoidance retention test indicative of an emotional memory deficit [16].

Our analysis of basal and swim stress-induced activation of the HPA axis revealed that p11KO mice exhibit increased ACTH and corticosterone levels. We have identified p11 expression in the parvocellular subnucleus of the PVN, where it is highly co-localized with AVP-producing neurons. AVP is known to be released somatodendritically in the PVN [50]. AVP cells in the PVN are sexually dimorphic and express high levels of the estrogen receptor β [51–54]. Given that there are higher levels of estrogen in female mice, the physiological responses of these cells show sex differences which may be exaggerated in p11KO mice. However, there does not seem to be a direct link between estrogen/estrogen receptor β signaling and p11 levels since we found no sexual dimorphism of p11 expression at the PVN. Blockade of V_1B_, but not CRH_1_, receptors prevented the post-stress hypersecretion of ACTH in p11KO mice. Higher ARC mRNA expression in the PVN of p11KO mice derived from AVP negative and CRH positive cells. Moreover, we confirmed a previously reported [37–39] V_1B_ expression in CRH positive cells of the PVN. We also found a broad expression of 5-HT_1B_ receptor transcripts in the PVN. Since p11 increases the cell surface levels of 5-HT_1B_ receptors and potentiates its inhibitory action [12, 55], we propose that lower cell surface levels of inhibitory 5-HT_1B_ receptors in p11KO may lead to higher somatodendritic AVP release rates and subsequent activation of ARC mRNA in CRH positive cells, through excitatory V_1B_ receptors. We cannot exclude that there are neuronal populations outside the PVN that co-express p11 and AVP which could contribute to our results. Further, it remains to be studied whether p11 can directly interact with V_1B_ receptors and stimulate their functionality in a way reflected by our results. It should also be noted that while the 5-HT_1B_ receptor/p11 complex may exert somatodendritic actions [56], it is more likely to play an important role in regulating neurotransmission at nerve terminals [12]. AVP neurons in PVN are glutamatergic and the 5-HT_1B_ receptor/p11 complex has been reported to regulate excitatory neurotransmission [12]. It is therefore possible that an increased glutamate release from AVP neurons in p11KO mice could contribute to our results, not least the behavioral phenotype. It has, indeed, been demonstrated that anxiety-like behaviors dependent on CRH cells rather relies on an excitatory glutamatergic projection to a subset of neurons in the perifornical region of the lateral hypothalamus than on endocrine signaling [57]. It is possible that excitatory transmission from AVP-secreting neurons plays a similar role in regulating stress-related behaviors.

In addition to the observed HPA dysfunction, the present study also provides clear evidence for SAM dysfunction, with no evidence of a correlation between the two axes. We show that p11KO mice have higher levels of adrenal catecholamines, though p11 was not detected in the catecholaminergic cells of adrenal medulla. Furthermore, the fact that SERT-p11cKO mice showed identical levels of adrenal epinephrine to the global p11KO indicate that epinephrine was modulated by changes in the activity of serotonergic neurons of the RMG, where p11 and 5-HT_1B_ transcripts are enriched. IML neurons receive inputs from the RMG and express both 5-HT_2A_ and 5-HT_2C_ receptors [41, 58, 59], denoting an excitatory role of serotonin upon the SAM axis. This suggests that lack of p11 in RMG cells may abolish the autoinhibitory mechanism of serotonin through local 5-HT_1B_ receptor downregulation and subsequently enhance the 5-HT_2A/2C_-induced IML activation. The sexually dimorphic characteristics, in terms of morphology and number, of IML cells [60] may account for the sex difference in epinephrine we observed. Interestingly, adrenal norepinephrine was over 50% higher in p11KO mice of both sexes but was unaltered in SERT-p11cKO mice. Thus, adrenal norepinephrine accumulation is p11-dependent but we show that it is not mediated by serotonergic neurons. A possibility is actually that norepinephrine accumulation may be dependent on the hyperexcitation of IML neurons that express 5-HT_1B_ receptors and p11.

These data showing hyperactivity of the SAM axis are consistent with physiological data on the HR. IML neurons project to sympathetic ganglia and adrenal medulla in order to subsequently regulate cardiovascular function [61, 62]. Telemetric ECG measurements revealed enhanced cardiovascular responsiveness in p11KO mice associated with a decreased HR variability by conditioned tone-dependent fear in the home cage, indicating hyperresponsiveness of the autonomic nervous system. Norepinephrine is an important regulator of vascular tone due to its high affinity for α1-adrenergic receptors. At the same time, epinephrine is characterized as crucial controller of cardiac output through its selectivity for cardiac β1-adrenergic receptors. The higher HR and lower HR variability observed in p11KO compared to WT mice may therefore be explained by epinephrine-dependent, β1-adrenergic receptor-mediated, mechanisms. The slower recovery of the sympathetic activation in p11KO mice is consistent with reports of reduced HR variability in anxiety disorders [63] based on delayed return of vagal function. An additional mechanism could be that p11 alters the intrinsic excitability of the heart through 5-HT_4_ receptors [64].

Taken together, our findings provide strong evidence that p11 deficiency leads to increased stress reactivity along with HPA and SAM axes hyperresponsiveness. By using pharmacological tools and SERT-p11cKO mice, we initiated the delineation of the circuitries underlying the observed hyperresponsiveness of the HPA and SAM axes. Our data indicate that p11 regulates the HPA axis through modulation of AVP cell activity at the PVN. A major task for future work will be to elucidate how p11 effector proteins, such as 5-HT_1B_, 5-HT_4_, and mGluR5 receptors and/or various ion channels [14], modulate PVN as well as HPA and SAM axes.

## Supplementary information

Supplementary Material
