# Supplementary material for: P11 deficiency increases stress reactivity along with HPA axis and autonomic hyperresponsiveness
Source: Mol Psychiatry. 2020 Oct 1;26(7):3253–65. doi: 10.1038/s41380-020-00887-0 (PMC8505237; doi:10.1038/s41380-020-00887-0)
Supplement: Supplementary file 1 — Supplementary Material [file 41380_2020_887_MOESM1_ESM.pdf]

P11 deficiency increases stress reactivity along with HPA axis and autonomic hyperresponsiveness

Sousa VC, Mantas I, Stroth N, Hager T, Pereira M, Jiang H, Jabre S, Paslawski W, Stiedl O, Svenningsson P

SUPPLEMENTARY INFORMATION

Supplementary Methods

Animals

Wild-type (WT), constitutive p11 knockout (p11KO) and conditional knockout of p11 in SERT expressing neurons (SERT-p11cKO), on a C57BL/6J background, were generated as previously described [1, 2], and bred at the animal facility of Karolinska Institutet. Mice were housed in Type III Macrolon cages, with *ad libitum* access to food and water, in a temperature- and humidity-controlled room under a regular 12-h light/dark cycle (lights on at 07:00 h). All experiments were approved by the Karolinska Institutet Ethical Committee and the VU University Amsterdam according to Swedish and Netherlands guidelines, respectively, in full compliance with European requirements.

Pup ultrasonic vocalizations:

Littermate mice from homozygous breeding pairs were subjected to unpredictable maternal separation combined with unpredictable maternal stress (MS; WT: n=4, p11KO: n=5) for 3 h daily, from postnatal day 1 to 14 (PND 1-14) [3], or were left completely undisturbed (control; WT n=8, p11KO n=7). The timing of separation was always within the light period, and was scheduled day to day in a randomized fashion. During separation, pups

were removed from their home cage and placed in clean cages, with cardboard separators between each littermate. Electrically heated pads maintained pup cage temperature at 33±1°C. Each individual dam was placed in a separate clean cage containing food and water. For 15 min, within the separation period, maternal stress consisted of placing the dam under restraint in a Decapicone (Braintree, MA, USA), or in a novel cage under stroboscopic illumination (~300 flashes/min). The maternal stressor applied each day was randomly picked for unpredictability.

Ultrasonic vocalizations were recorded over a period of 5 min from pups of both sexes, on PND 3, 6, 9 and 12. Pups were placed on top of a clean paper towel inside the recording chamber. The recording chamber consisted of a clean cage covered with acoustic foam on the outside and was kept at a temperature of 27±1°C. Ultrasonic calls were recorded using a condenser microphone connected to the Avisoft Ultra Sound Gate 116Hb system (Avisoft Bioacoustics, Glienicke, Germany). The number of ultrasonic calls was quantified using the Avisoft real-time monitoring tool. Pups undergoing MS (WT: n=28, p11KO: n=29) were taken for recording after removal of the dam during the separation period. Non-stressed littermate pups (control, WT: n=47, p11KO: n=34) were allowed 2 h of acclimatization to the testing room before start of recordings. Thereafter, pups were carefully and individually removed only for the duration of the recording, otherwise leaving littermates and dams undisturbed in a separate, ventilated, soundproof cage cabinet. Pups were weaned at PND21 and left undisturbed until 10 weeks of age, when behavioral testing began.

Maternal behavior:

Genotype differences in maternal behavior were studied in a set of 8 WT and 7 p11KO litters kept under normal rearing conditions. This was done by measuring nesting behavior at PND4-13 and assessing performance in the pup retrieval test at PND5 and PND7 [4]. For nest

building measurements, litter nests were carefully removed, and a wad of pure cotton weighing 2 g was placed in the food tray. Since all cotton pulled into the cage by the mice was added to the nest, the percentage of the provided nesting material used in a 24-h period was considered a reliable measure of nest construction [4]. In the pup retrieval test, pups were removed from the home cage for 5 min, and placed in a separate clean cage kept at 33±1°C under an infrared heat lamp. Four pups were scattered in the quadrant opposite to the nest and the time the mother retrieved the first and the last pup was recorded within a 10-min period. Nesting behaviors and licking/grooming were also manually scored during the observation period.

Adult behavioral phenotyping:

We found no rearing-induced differences in adult behavioral phenotype (data not shown), therefore animals from the MS groups seen in Figure 1a were excluded from the subsequent adult phenotyping results and statistics. Resilience to maternal stress was previously reported in some mouse strains, including C57BL/6, whose dams are characterized as providing better care to their pups following periods of deprivation [5, 6]. This suggests that the gene × environment interaction observed in early postnatal age was dissipated by overcompensation of maternal care. Nevertheless, it is also evident that the response of C57BL/6 pups to maternal care and the life-long consequences of p11 deficiency are mutually exclusive.

Mice were tested at one-week intervals, i.e. testing took place over four consecutive weeks and tests took place at identical times of the day, to avoid circadian-dependent changes.

Open field test:

Mice were placed in a 55 cm x 55 cm squared box for measurement of locomotion during a trial duration of 5 minutes. Total distance travelled, average speed and time spent in the

centre zone were measured using an automated video tracking system (Noldus Ethovision XT 11, Wageningen, Netherlands).

Post-shock vocalizations and passive avoidance test:

Mice were placed in the light compartment of a step-through passive avoidance apparatus (Ugo Basile, Comerio-Varese, Italy) for 60 s, before a sliding door was opened and the mouse entered a dark compartment (training latency). After the animal stepped into the dark compartment with all four paws, the door automatically closed and a weak electrical stimulus (0.3 mA, 2 s duration, scrambled current) was delivered through the grid floor. Immediately post-shock, mouse vocalizations were recorded for 5 s and the number of vocalizations emitted as well as vocal amplitude were measured using Avisoft Ultra Sound Gate Analyzer (Avisoft Bioacoustics, Glienicke, Germany). After a 24-h delay, a mouse was again placed in the light compartment and the step-through latency to return to the dark compartment was measured (retention latency).

Light-dark exploration test:

The light-dark exploration test was conducted as previously described [7]. The cumulative time spent by mice in a brightly illuminated vs. dark compartment over a 15-min session was detected and analyzed using an automated video tracking system (Noldus EthoVision XT 8, Wageningen, The Netherlands).

Response to acute swim stress, serum collection and quantification of hormones:

Testing took place between 09:00 and 12:00 for all sets of all cohorts, to ensure that no circadian variation in hormones confounded the results. Mice were placed into 50 cm tall tanks filled with water (temperature 24±1°C) to 20 cm depth with 7-min test duration. After removal from the water tank, mice were either gently dried and placed in their home cages

lined with dry paper towels for a 24-min (Supplementary Figure 3) rest, or were immediately (within 1 min, all other stress conditions) euthanized by decapitation. Immobility in the forced swim test was analyzed in one of the cohorts from video recordings using Noldus EthoVision.

For pup ACTH analysis, we collected blood samples from a separate set of non-stressed litters than the one used for USV recordings. Pups were taken from homozygous breeding pairs (WT: n=13, p11KO: n=15) at PND3, 6, 9 or 12. Sex was not differentiated given that it is widely accepted that ACTH is not sexually differentiated in this developmental period. Litters were gently moved into a separate cage (kept at 33±1°C by electrically heated pads) to which some of the litter home cage bedding material was added. Trunk blood was quickly collected by decapitation. To meet the minimum assay volumes, each sample of trunk blood had to be pooled from 6 (PND3), 4 (PND6), or 3 (PND9 &12) pups.

Serum collection, ACTH and corticosterone assays were conducted as previously described [8]. ACTH (MD Biosciences, St. Paul, MN, USA) and corticosterone (Enzo Life Sciences, Farmingdale, NY, USA) were measured using commercially available ELISA kits, following the manufacturer’s instructions. Serum samples were used undiluted (ACTH) or diluted 1:10 in assay buffer (corticosterone).

Tissue collection, RNA extraction, reverse transcription and quantitative PCR:

Immediately following blood collection, brains were quickly removed, snap frozen in isopentane (Sigma-Aldrich, St. Louis, MO, USA) cooled in dry ice, and subsequently stored at -80°C. Adrenal glands were also dissected, frozen in dry ice and stored at -80°C.

In a cryostat chamber kept at -20°C, one 2 mm thick coronal slice (0.0 to -2.0 mm from bregma) was cut from each brain using a 0.5 mm interval mouse brain matrix (AgnThos, Lidingö, Sweden). A tissue piece of approximately 2 x 2 x 2 mm (width x height x thickness) generously encompassing the paraventricular nucleus (PVN) of the hypothalamus was excised

from this slice using a scalpel blade. The tissue pieces were immediately placed on dry ice and then kept at -80°C until RNA extraction.

Hypothalamic tissue samples were disrupted in lysis buffer (RNeasy Mini kit, Qiagen, Hilden, Germany) using an ultrasonic processor (EpiShear Probe Sonicator, Active Motif, La Hulpe, Belgium). Total RNA was subsequently extracted from the lysates (RNeasy Mini kit, Qiagen) and samples were treated with RNase-free DNase I (Thermo Scientific, Stockholm, Sweden). Per sample, an aliquot of 0.5 µg DNase-treated RNA was primed with random hexamers and subjected to reverse transcription (RevertAid H Minus First Strand cDNA Synthesis Kit, Thermo Scientific). Quantitative PCR was carried out in a total reaction volume of 22 µl, containing 2 µl cDNA (each sample assayed in duplicate), 1X Power SYBR Green Master Mix (Life Technologies, Stockholm, Sweden) and 230 nM gene-specific primers (CyberGene, Solna, Sweden). Primer sequences were as follows: Avp-F, GCCAGGATGCTCAACACTACG; Avp-R, TCTCAGCTCCATGTCAGAGATG; Crh-F, CTCAGCAAGCTCACAGCAAC; Crh-R, GGAGCTGCGATATGGTACAGA (where F and R denote forward and reverse primers, respectively). Reactions were run on a StepOnePlus Real-Time PCR System (Life Technologies) together with a standard curve of known cDNA concentrations for each target gene. After an initial DNA denaturation and polymerase activation step (10 min at 95°C), 40 cycles of denaturation (15 s at 95°C) and annealing/extension (1 min at 55°C) were carried out. Raw data were analyzed using StepOne Software (version 2.2.2) and "Quantity Mean" values (corresponding to abundance of target mRNA) were exported to Excel (Microsoft; Redmond, WA, USA). Finally, all values were converted to "% WT control" prior to statistical analysis.

In-situ hybridization (ISH):

For the experiments in Figure 2g-h, whole brains from WT and p11KO mice were collected at baseline (control) or 15 min after an acute swim stress trial of 7-min duration (post-stress). For the experiments using fluorescent *in-situ* hybridization (FISH), whole brains, pituitary glands, adrenal glands, heart, and spinal cord (T2–L2 segments), were taken from unstressed WT, p11KO, or SERT-p11cKO mice. Tissue was snap frozen in isopentane, cooled in dry ice, and subsequently stored at -80°C. Coronal sections (14 µm thick) were collected at -0.94 mm and -1.06 mm from Bregma using a cryostat sectioner (Leica, Kista, Sweden), and thaw-mounted on Polysine® glass slides (VWR, Stockholm, Sweden). Sections were fixed in 4% paraformaldehyde (Sigma-Aldrich, St. Louis, MO, USA) and hybridized with ^{35}S-radiolabeled antisense riboprobes against p11, and activity-regulated cytoskeleton protein (arc), as previously described [1, 9]. After hybridization, slides were exposed to Kodak Biomax maximum resolution film (Carestream, Rochester, NY), in room temperature for 21 days prior to development. Densitometric measurements of mRNA expression were obtained from digitized autoradiograms using the NIH ImageJ 1.40 software (National Institute of Mental Health, Bethesda, MD, USA) and are presented as optical density values. The brain areas were selected according to *The mouse brain in stereotaxic coordinates* [10].

Fluorescent ISH (RNAscope) were performed using the RNAscope Multiplex Fluorescent Assay (Advanced Cell Diagnostics, Abingdon, Oxford). Cryostat (Leica CM 3050 S) fresh frozen sections (12 µm thick) were post fixed in 4% PFA for 15 min at 4°C and dehydrated in graded alcohols. Afterwards, it was applied Protease IV (Advanced Cell Diagnostics) for 30 min at room temperature. Sections were then hybridized with the probes: *p11* (Mm-S100a10, cat. 410901), *5-HT$_{1B}$* (Mm-Htr1b-C3, cat. 478901-C3), CRH (Mm-Crh-C2, cat. 316091-C2), *AVP* (Mm-Avp-C3, cat. 401391-C3), *Avpr1b* (Mm-Avpr1b , cat. 480141), *ARC* (Mm-Arc, cat. 316911), *Tph2* (Mm-Tph2-C2, cat. 318691-C2) and *Th* (Mm-Th-C2,

cat. 317621-C2) for 2 h at 40 °C. The hybridization step was followed by standardized steps of amplification (Amp 1-FL 30 min at 40 °C, Amp 2-FL 15 min at 40 °C, Amp 3-FL 30 min at 40 °C, Amp 4C-FL 15 min at 40 °C). After the last amplification step, sections where counterstained with DAPI (Advanced Cell Diagnostics), or immunostained with a polyclonal ChAT primary antibody (catalogue# AB144P, Millipore, Solna, Sweden), and an anti-goat Alexa Fluor 568 secondary (Invitrogen, Stockholm, Sweden) , then mounted with Dako fluorescent mounting medium (Agilent Technologies, Kista, Sweden). Sections were imaged on a Carl Zeiss LSM 880 confocal microscope (Carl Zeiss AB, Stockholm, Sweden) using either a 20x objective or a 63× oil immersion objective. Z-stacks of 7-10 µm thickness were obtained in each caption. Quantification of cell expression of p11, 5-HT_{1B}, CRH and AVP was calculated from the total cell counts from $\geq$ 4 image captures of 6-5 stacks at 63× magnification, from PVN section of n=3 WT mice of each sex. We took only sections from females at preestrus, which we controlled through aspiration of vaginal fluid and observing the morphology of epithelial cells in a light microscope.

High-performance liquid chromatography:

Catecholamine content of mouse adrenal glands was measured by high-performance liquid chromatography (HPLC), using a method based on previously published procedures [11, 12]

<u>Chemicals</u>

L-Norepinephrine hydrochloride (NE), (±)-Epinephrine hydrochloride (EPI), acetonitrile (Chromasolv Plus), monobasic sodium phosphate, ethylene-diamine-tetra-acetic acid (EDTA) disodium salt, 1-octanesulfonic acid (OSA) sodium salt, triethylamine (TEA), 85% phosphoric acid, 70% perchloric acid (PCA), and sodium bisulfite were purchased from Sigma-Aldrich (Stockholm, Sweden). HPLC-grade water was produced using a Milli-Q Ultra-Pure water system (resistance 18.2 MΩ·cm, Billerica, MA, USA).

High performance liquid chromatography

The HPLC-ECD system was a Dionex Ultimate 3000 series (all parts from Dionex, ThermoFisher Scientific, Stockholm, Sweden) which consisted of an ISO-3100BM HPLC pump, WPS-3000TBSL analytical autosampler with temperature control, SRD-3200 solvent rack with built-in 2-channel vacuum degasser, Coulochem III detector with thermal organizer module, and a 5011A coulometric analytical cell.

Analyte separation was performed on a Dionex C18 reversed-phase MD-150 3.2 mm x 150 mm, 3 µm column. Column and analytical cell were kept at 37°C. The mobile phase, which was pumped at a flow rate of 0.4 mL/min, consisted of 75 mM monobasic sodium phosphate, 1.7 mM OSA, 100 µL/L TEA, 25 µM EDTA, and 10% acetonitrile (v/v), and filtered through a 0.2 µm nylon membrane. The pH was adjusted to 3.0 (Jenway Model 3510 pH meter, Gothenburg, Sweden) with 85% phosphoric acid, degassed for approximately 5 minutes, and equilibrated overnight on the HPLC-ECD system. The first and second analytical cells were set to -100 mV and +300 mV, respectively. Processed samples were thawed on ice about an hour before analysis, placed in the autosampler, and kept at 5°C before injection. The injection volume was 20 µL. The acquisition time was 15 minutes on the column. Chromatograms were acquired with Dionex Chromeleon 7 software.

Preparation of tissue samples

Both adrenal glands were quickly excised immediately following blood collection from each mouse, transferred to polypropylene tubes, immediately frozen on dry ice and then transferred to storage at -80˚C.

Adrenal glands were suspended in 500 µL ice-cold 0.1 M PCA and homogenized immediately using an ultrasonic processor (EpiShear™ Probe Sonicator, Active Motif, La Hulpe, Belgium) with a 3 mm probe at 50% amplitude for 6 seconds, until complete tissue disruption. All

tissue samples were processed in random order. Samples were incubated on ice for 10 minutes, vortexed, and centrifuged at 16,000 x g for 15 minutes at 4°C. The supernatants were transferred to spin columns with 0.2 µm nylon membrane filters and centrifuged at 16,000 x g for 2 minutes.

Adrenal eluates were diluted 1:100 in 0.1 M PCA and immediately stored at -80°C. All samples were injected into the HPLC within two weeks.

Standard solutions and calibration

Standard solutions of 500 µg/mL NE and EPI were stored at -80°C as aliquots in 0.1 M PCA and 5 mM sodium bisulfite. A standard mix solution containing both standards was prepared in 0.1 M PCA with a concentration of 10 µg/mL per standard. This standard mix solution was further diluted with 0.1 M PCA to obtain final calibration concentrations of 10, 50, 100, and 200 ng/mL. The calibration curves were obtained with the Chromeleon software by using linear regression of peak area versus concentration. Coefficients of determination were 0.9988 or higher. Analyte concentrations in tissue samples were expressed as ng/ml and then converted to percent of wild-type.

Radio-telemetry, novelty exposure and auditory trace fear conditioning:

Adult male mice were implanted with an ECG radio-transmitter and subjected to novelty exposure and to auditory trace fear conditioning with a 30-s trace interval and a 2-s shock of 0.7 mA (constant current) to determine HR responses indicative of unconditioned and conditioned fear, respectively [13]. ECG radio-transmitters (ETA-F10, Data Sciences International, St. Paul, MN, USA) were implanted intraperitoneally as described before [14]. ECG was remotely measured via a receiver board (RLA1020, Data Sciences International), digitally recorded (4 kHz sampling rate) and analyzed offline using Chart software (v7.1, ADInstruments, Spechbach, Germany). The intervals between successive R-peaks of the ECG

complex served as inter-beat intervals to determine instantaneous HR (given in beats per min; bpm). ECG signals were digitally recorded at 4 kHz sampling rate. HR variability was calculated on the basis of the root-mean-square of subsequent heart beat interval differences (RMSSD, given in ms) [14]. After a recovery period of 2-3 weeks mice were subjected to novelty exposure for a total of 34 min as reported before [14]. Three days later the mice were trained in auditory trace fear conditioning. Auditory trace fear conditioning was performed with a 30-s trace interval, since this trace interval renders auditory fear conditioning hippocampus-dependent [15]. Thereafter, retention and extinction of conditioned fear to the auditory cue was determined in the home cage of mice at 24-h interval for 5 days. All experiments occurred during the light phase, when locomotor activity is low and does not contribute to autonomic effects.

Experimental design and Statistical analysis

The sample sizes were based on previous reports to ensure adequate power. The experimenter was blinded to group allocation during experiment, and analysis was automated. Animals were pseudo-randomly allocated to experimental groups, so that each cage would contain balanced group distribution. Outliers in the data were calculated using the Grubb's test calculator tool from GraphPad (GraphPad, San Diego, CA, USA), and removed. Normality of the data distribution was checked with a Normal probability plot (InVivoStat program [16]). Statistical analysis was carried out by two-way or three-way analysis of variance (ANOVA) or repeated measures ANOVA followed by Fisher's least significance difference (LSD) post-test, where indicated, using the InVivoStat [16] program. All data were plotted using GraphPad Prism 8 (GraphPad, San Diego, CA, USA) All data is presented as mean ± standard error of the mean (SEM) of the number of animals per group detailed in Supplementary Table 1.

Supplementary results

Pup ACTH and Maternal behavior

To check whether the greater number of vocalizations seen in non-stressed p11KO mice reflects the HPA activity, we took trunk blood samples under non-stressed (control) conditions from a separate set of litters and saw that ACTH levels do not reflect the pups USVs (Supplementary Fig. 1a). This finding is in line with evidence that USVs are negatively correlated with plasma ACTH concentrations under the stress hyporesponsive period [17], and indicates that the USVs are a behavioral response differentially regulated from HPA axis activity at this stage of development.

Mouse maternal behavior is strongly influenced by pup calls in the ultrasonic range reviewed in [18, 19]. We therefore used an undisturbed set of litters (normal rearing conditions) to investigate maternal behavior of p11KO versus WT mice. We first evaluated nesting behavior between PND4-13 by measuring the amount of nesting material used over 6 periods of 24 h (Supplementary Figure 1b). Compared to WT dams (77±7%, n=8), p11KO dams consistently showed near-complete usage of nesting material (99±1%, n=7, $p<0.05$, Student's t-test). Maternal behavior in the pup retrieval test was also evaluated at PND5 and PND7 (Supplementary Figure 1c-e). In the observation period of this test, indices of nesting behavior (Supplementary Figure 1c) and licking/grooming (Supplementary Figure 1d) were slightly higher in p11KO dams ($p>0.05$) while there were no genotype differences in latency to pup retrieval (Supplementary Figure 1e). Overall, we found no evidence for impaired maternal behavior in p11KO mice. In fact, some aspects of maternal behavior appear to be enhanced in p11KO compared to WT mice, perhaps as a consequence of enhanced USVs from p11KO pups (see above). This in turn suggests that exacerbated separation distress in p11KO pups is driven by endogenous factors rather than poor maternal behavior.

Mobility in the Open Field (OF) test

Measurements of mobility in the open field show that there is an overall higher mobility in the p11KO mice, as indicated by significantly higher pathlength (Supplementary Figure 2a) and average speed (Supplementary Figure 2b) in p11KO females, compared to wildtypes. However, we did not see a significant difference in zone transitions (data not shown). Since we also see that p11KO mice have a significantly higher activity when placed in a novel environment (Figure 4f), results suggest that the higher mobility in p11KO may be a novelty-induced arousal reaction.

Serum corticosterone levels in p11KO

We compared hormone concentrations at baseline (non-stressed, control), 1-min post-stress and 24-min post-stress (Supplementary Figure 3). Female p11KO mice had approximately 3-fold higher baseline corticosterone when compared to that of WT mice. There was no effect of genotype on basal corticosterone secretion in male mice.

Swim stress increased circulating levels of corticosterone, at 1 min post-stress, in both genotypes and sexes. Female p11KO mice showed approximately 50% higher corticosterone level than WT mice 1 min post-stress. Corticosterone level at 24 min post-stress was 70% higher in female p11KO mice than WT mice (insert in Supplementary Figure 3).

Interestingly, corticosterone concentrations in male p11KO mice were the same as in WT at 1 min post-stress, but 50% higher than WT after a 24-min post-stress resting period (p=0.054; insert in Supplementary Figure 3). Overall, compared to WT, male p11KO mice thus appear to display enhanced HPA axis activity in response to forced swimming, possibly in the form of delayed recovery. On the other hand, female p11KO mice exhibit HPA axis overdrive in the absence of an acute stressor, as well as post-stress hyperreactivity.

Expression of V_{1B} in the CA2 area of the hipocampus

We performed fluorescent *in situ* hydridization (FISH, RNAscope), with the AVP V_{1B} receptor probe, in the hippocampus of WT mouse. V_{1B} receptors are known to be highly expressed in the CA2, but not other areas, of the hippocampus [20]. Therefore, we validated the specificity of the signal we observed in the PVN (Figure 2n) by performing FISH in a section of the hippocampus (Supplementary Figure 4). We observed strong expression of V_{1B} mRNA only in the CA2, thus confirming the specificity of the V_{1B} signal we observed in CRH positive cells of the PVN.

Selective p11 deletion in DR

P11 is highly expressed in most of the serotoninergic cell groups, such as B7 (dorsal raphe, DR), and B3 (raphe magnus nucleus, RMG), where it is highly co-localized with 5-HT_{1B} receptors (Supplementary Figure 5a-c). The serotonergic system is implicated in the regulation of both HPA and SAM axis response. For this reason, we used a Cre-lox system to knock out p11 in the serotonin transporter (SERT) positive cells, the SERT-p11cKO. For validation of suppression of p11 expression in serotonergic cells we used both radioactive and double fluorescent in situ hydridization (RNAscope) with p11 probes (Supplementary Figure 5d-i). To confirm the selective suppression of p11 to serotonergic neurons used the Tph2 mRNA as a marker (Supplementary Figure 5b, c). The loxP sites for the p11 suppression are located in either side of exon 2 while the ZZ p11 probe hybridizes a region from exon 1 to exon 3 (Supplementary Figure 5d,g). For this reason, there is no complete loss but a robust reduction of both radioactive and fluorescent signals (Supplementary Figure 5h,i). However, no p11 protein is encoded.

Norepinephrine and epinephrine levels presented as ng/mg adrenal tissue

Supplementary Figure 6 shows absolute measures of adrenal norepinephrine and epinephrine levels normalized against mg adrenal tissue. In each experimental run, epinephrine levels were higher than the norepinephrine levels, but the magnitude differed. To facilitate comparisons from these distinct experimental runs, we present these data as percent of WT vehicle/control in Figure 3 of the main paper. Figure 3 also indicates relevant statistical analyses.

P11 expression in cardiac tissue

We performed fluorescent in situ hydridization (RNAscope) with a p11 probe to detect its expression in WT mouse cardiac tissue (Supplementary Figure 7). Previous studies showed that p11 regulates cardiomyocyte calcium through 5-HT_4 receptors [21]. We detected low amounts of p11 mRNA in cardiac tissue (Supplementary Figure 7).

Supplementary References

1. Svenningsson P, Chergui K, Rachleff I, Flajolet M, Zhang X, El Yacoubi M *et al.* Alterations in 5-HT1B receptor function by p11 in depression-like states. *Science* 2006; **311**(5757)**:** 77-80.

2. Schintu N, Zhang X, Alvarsson A, Marongiu R, Kaplitt MG, Greengard P *et al.* p11 modulates L-DOPA therapeutic effects and dyskinesia via distinct cell types in experimental Parkinsonism. *Proc Natl Acad Sci U S A* 2016; **113**(5)**:** 1429-1434.

3. Franklin TB, Russig H, Weiss IC, Graff J, Linder N, Michalon A *et al.* Epigenetic transmission of the impact of early stress across generations. *Biol Psychiatry* 2010; **68**(5)**:** 408-415.

4. Lonstein JS, Fleming AS. Parental behaviors in rats and mice. *Curr Protoc Neurosci* 2002; **Chapter 8:** Unit 8 15.

5. Savignac HM, Dinan TG, Cryan JF. Resistance to early-life stress in mice: effects of genetic background and stress duration. *Front Behav Neurosci* 2011; **5:** 13.

6. Millstein RA, Holmes A. Effects of repeated maternal separation on anxiety- and depression-related phenotypes in different mouse strains. *Neurosci Biobehav Rev* 2007; **31**(1)**:** 3-17.

7. Kiselycznyk C, Zhang X, Huganir RL, Holmes A, Svenningsson P. Reduced phosphorylation of GluA1 subunits relates to anxiety-like behaviours in mice. *Int J Neuropsychopharmacol* 2013; **16**(4)**:** 919-924.

8. Stroth N, Liu Y, Aguilera G, Eiden LE. Pituitary adenylate cyclase-activating polypeptide controls stimulus-transcription coupling in the hypothalamic-pituitary-adrenal axis to mediate sustained hormone secretion during stress. *J Neuroendocrinol* 2011; **23**(10)**:** 944-955.

9. Eriksson TM, Delagrange P, Spedding M, Popoli M, Mathe AA, Ogren SO *et al.* Emotional memory impairments in a genetic rat model of depression: involvement of 5-HT/MEK/Arc signaling in restoration. *Mol Psychiatry* 2012; **17**(2)**:** 173-184.

10. Paxinos G, Franklin KBJ. *The mouse brain in stereotaxic coordinates*. Compact 2nd edn. Elsevier Academic Press: Amsterdam ; Boston, 2004.

11. Hubbard KE, Wells A, Owens TS, Tagen M, Fraga CH, Stewart CF. Determination of dopamine, serotonin, and their metabolites in pediatric cerebrospinal fluid by isocratic high performance liquid chromatography coupled with electrochemical detection. *Biomed Chromatogr* 2010; **24**(6)**:** 626-631.

12. Yang L, Beal MF. Determination of neurotransmitter levels in models of Parkinson's disease by HPLC-ECD. *Methods Mol Biol* 2011; **793:** 401-415.

13. Stiedl O, Jansen RF, Pieneman AW, Ogren SO, Meyer M. Assessing aversive emotional states through the heart in mice: Implications for cardiovascular dysregulation in affective disorders. *Neurosci Biobehav Rev* 2009; **33**(2)**:** 181-190.

14. Hager T, Maroteaux G, du Pont P, Julsing J, van Vliet R, Stiedl O. Munc18-1 haploinsufficiency results in enhanced anxiety-like behavior as determined by heart rate responses in mice. *Behav Brain Res* 2014; **260:** 44-52.

15. Misane I, Tovote P, Meyer M, Spiess J, Ogren SO, Stiedl O. Time-dependent involvement of the dorsal hippocampus in trace fear conditioning in mice. *Hippocampus* 2005; **15**(4)**:** 418-426.

16. Bate ST, Clark RA. *The design and statistical analysis of animal experiments*. Cambridge University Press: Cambridge, 2014, 310pp.

17. Sapolsky RM, Meaney MJ. Maturation of the adrenocortical stress response: neuroendocrine control mechanisms and the stress hyporesponsive period. *Brain Res* 1986; **396**(1)**:** 64-76.

18. Scattoni ML, Crawley J, Ricceri L. Ultrasonic vocalizations: a tool for behavioural phenotyping of mouse models of neurodevelopmental disorders. *Neurosci Biobehav Rev* 2009; **33**(4)**:** 508-515.

19. Noirot E. Ultrasounds and maternal behavior in small rodents. *Dev Psychobiol* 1972; **5**(4)**:** 371-387.

20. Young WS, Li J, Wersinger SR, Palkovits M. The vasopressin 1b receptor is prominent in the hippocampal area CA2 where it is unaffected by restraint stress or adrenalectomy. *Neuroscience* 2006; **143**(4)**:** 1031-1039.

21. Meschin P, Demion M, Cazorla O, Finan A, Thireau J, Richard S *et al.* p11 modulates calcium handling through 5-HT(4)R pathway in rat ventricular cardiomyocytes. *Cell Calcium* 2015; **58**(6)**:** 549-557.

Supplementary Table 1 (continued in next page):

Group	n=	Seen in Figure	Cohort	Comments
Male WT	8	1b-d; insert in Supp. Fig 2	1	
Male KO	9	1b-d; insert in Supp. Fig 2	1	
Female WT	11	1b-d; insert in Supp. Fig 2	1	
Female KO	8	1b-d; insert in Supp. Fig 2	1	
MS WT (Mixed sex)	28	1a	1	non-separated controls in this cohort were completely undisturbed, thus did not undergo USV recordings
MS KO (Mixed sex)	29	1a	1	
WT Control (Mixed sex)	47	1a	2	
KO Control (Mixed sex)	34	1a	2	
WT Dams	8	Supp. Fig 1b-e	2	
KO Dams	7	Supp. Fig 1b-e	2	
Male WT Control	9	2e, j, k; Supp. Fig 2, 3	2	
Male KO Control	5	2e, j, k; Supp. Fig 2, 3	2	
Female WT Control	9	2e, j, k; Supp. Fig 2, 3	2	
Female KO Control	10	2e, j, k; Supp. Fig 2, 3	2	
Male WT Stress	8	1e; 2e, j, k; Supp. Fig 2, 3	2	
Male KO Stress	8	1e; 2e, j, k; Supp. Fig 2, 3	2	
Female WT Stress	9	1e; 2e, j, k; Supp. Fig 2, 3	2	
Female KO Stress	10	1e; 2e, j, k; Supp. Fig 2, 3	2	
Male Cre-/- Control	9	2q; 3i, j, Supp. Fig 6	3	
Male SERT-p11cKO Control	14	2q; 3i, j, Supp. Fig 6	3	
Female Cre-/- Control	8	2q; 3i, j, Supp. Fig 6	3	
Female SERT-p11cKO Control	6	2q; 3i, j, Supp. Fig 6	3	
Male Cre-/- Stress	10	2q; 3i, j, Supp. Fig 6	3	
Male SERT-p11cKO Stress	15	2q; 3i, j, Supp. Fig 6	3	

Female Cre-/- Stress	11	2q; 3i, j, Supp. Fig 6	3	
Female SERT-p11cKO Stress	10	2q; 3i, j, Supp. Fig 6	3	
Male WT Veh	9	2l; 3f, g, Supp. Fig 6	4	
Male KO Veh	7	2l; 3f, g, Supp. Fig 6	4	
Male WT CP	9	2l; 3f, g, Supp. Fig 6	4	
Male KO CP	7	2l; 3f, g, Supp. Fig 6	4	
Female WT Veh	9	2l; 3f, g, Supp. Fig 6	4	
Female KO Veh	7	2l; 3f, g, Supp. Fig 6	4	
Female WT CP	8	2l; 3f, g, Supp. Fig 6	4	
Female KO CP	8	2l; 3f, g, Supp. Fig 6	4	
Male WT Veh	8	2m; 3f, g, Supp. Fig 6	5	
Male KO Veh	8	2m; 3f, g, Supp. Fig 6	5	
Male WT TASP	9	2m; 3f, g, Supp. Fig 6	5	
Male KO TASP	10	2m; 3f, g, Supp. Fig 6	5	
Female WT Veh	9	2m; 3f, g, Supp. Fig 6	5	
Female KO Veh	7	2m; 3f, g, Supp. Fig 6	5	
Female WT TASP	12	2m; 3f, g, Supp. Fig 6	5	
Female KO TASP	8	2m; 3f, g, Supp. Fig 6	5	
Male WT Control	7	2f, g; 3d, e	6	
Male KO Control	12	2f, g; 3d, e	6	
Female WT Control	8	2f, g; 3d, e	6	
Female KO Control	7	2f, g; 3d, e	6	
Male WT Stress	8	2f, g; 3d, e	6	
Male KO Stress	11	2f, g; 3d, e	6	
Female WT Stress	8	2f, g; 3d, e	6	
Female KO Stress	9	2f, g; 3d, e	6	
Male WT	13	4a-l	7	
Male KO	14	4a-l	7	
PND3 WT (Mixed sex)	13	Supp. Fig 1a	8	each pooled from 6 pups
PND3 KO (Mixed sex)	14	Supp. Fig 1a	8	each pooled from 6 pups

PND6 WT (Mixed sex)	10	Supp. Fig 1a	8	each pooled from 4 pups
PND6 KO (Mixed sex)	14	Supp. Fig 1a	8	each pooled from 4 pups
PND9 WT (Mixed sex)	15	Supp. Fig 1a	8	each pooled from 3 pups
PND9 KO (Mixed sex)	17	Supp. Fig 1a	8	each pooled from 3 pups
PND12 WT (Mixed sex)	18	Supp. Fig 1a	8	each pooled from 3 pups
PND12 KO (Mixed sex)	13	Supp. Fig 1a	8	each pooled from 3 pups
Male WT	3	2d	9	
Female WT	3	2d	9	Confirmed at Preestrus

Supplementary Table 1. Number of subjects/samples per group

Supplementary Figures:

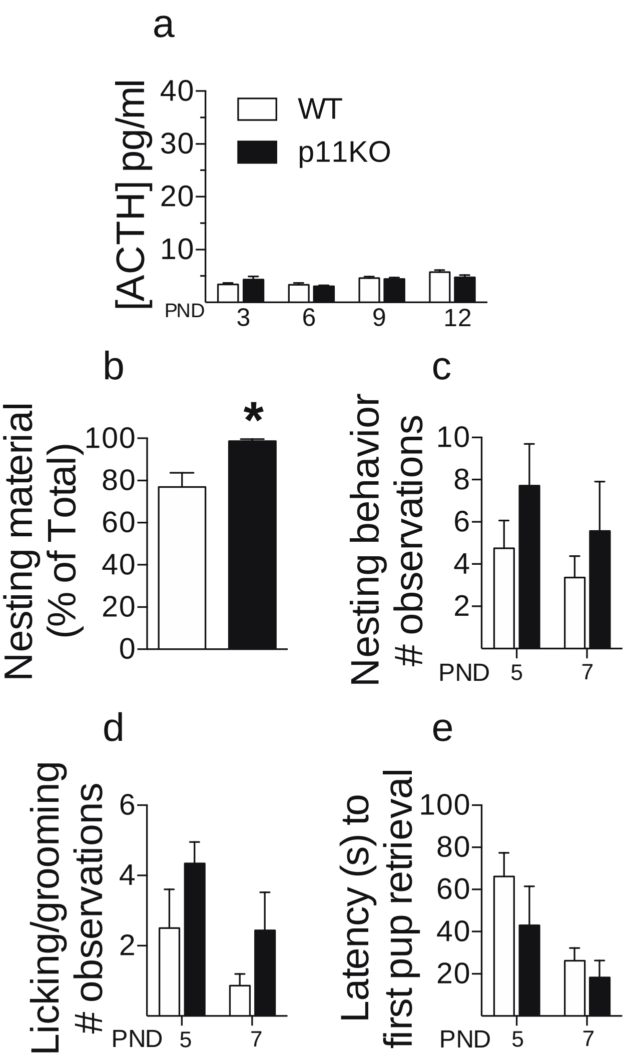

Supplementary Figure 1. Pup adrenocorticotropic hormone (ACTH) and maternal behavior. **(a)** Serum concentration of ACTH was measured in non-stressed p11KO and WT pups at PND3, 6, 9 and 12. Note that the low ACTH levels are in agreement with the expected stress-hyporesponsive period. ANOVA showed a significant age effect (p<0.0001, $F_{(3,106)}$=11.88), but no genotype differences. **(b)** Percentage of nesting material used by dams over 6 separate 24-h trials between PND4 and PND13 of their pups. Compared to WT, p11KO dams showed very consistent near-complete usage of nesting material. Nesting behavior **(c)**, licking/grooming **(d)**, and latency to first pup retrieval **(e)** were observed in a pup retrieval

test at PND5 and PND7. There was no evidence for altered maternal behavior in p11KO dams. All data represent mean ± SEM. *$p<0.05$, unpaired, two-tailed Student's *t*-test.

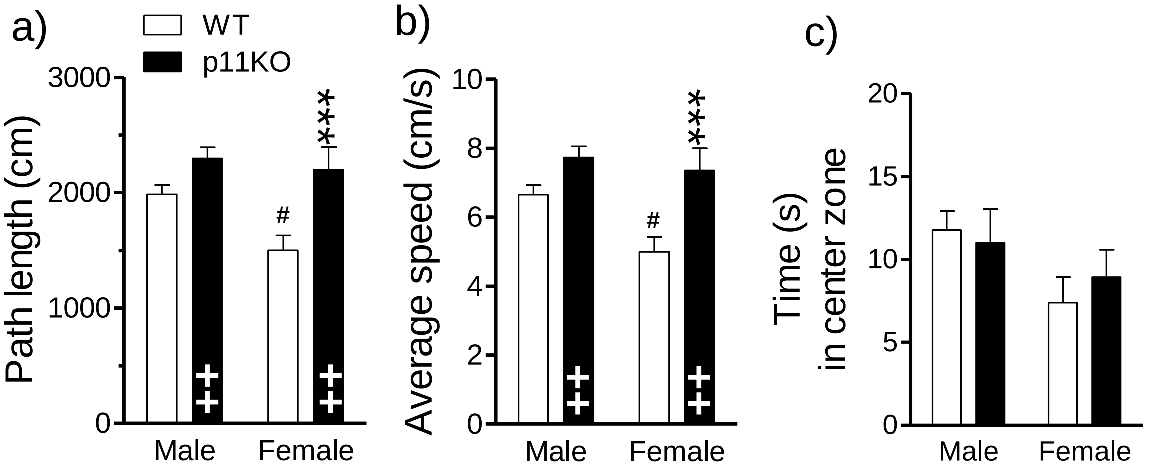

Supplementary Figure 2: Performance in the open field test. Genotype differences in **(a)** path length (p<0.01, $F_{(1,29)}$=9.75) and **(b)** speed (p<0.01, $F_{(1,29)}$=10.08), were stronger between female p11KO and WT mice (p<0.0001). Neither genotype nor sex had an effect on the time spent in the center zone of the open field **(c)**. Since between-zone transitions were not significantly higher in female p11KO mice (data not shown), this suggests that the higher mobility in p11KO females may be a novelty-induced arousal reaction. Data represent mean ± SEM of n≥8 per group. ^{++}p<0.01 overall genotype difference; ***p<0.001, versus corresponding WT; $^{\#}p$<0.05, versus male of same genotype; calculated with a two-way ANOVA followed by Fisher's LSD test.

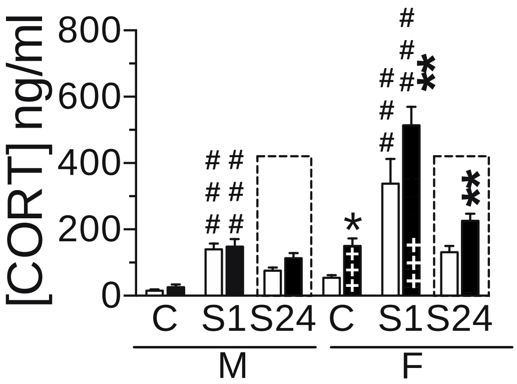

Supplementary Figure 3. Serum concentrations of corticosterone (CORT) were measured in male (M) and female (F) adult WT and p11KO mice at baseline (C), 1 min (S1) or 24 min (S24) post-swim stress. In females, there was a significant effect of genotype ($p<0.001$, $F_{(1,33)=}17.08$) and of stress (S1, $p<0.0001$, $F_{(1,33)=}48.94$). In males, there was only a significant effect of stress (S1, $p<0.0001$, $F_{(1,25)=}69.69$). The male p11KO corticosterone levels at S24 were 50% higher than in WT mice and showed a strong trend for difference ($p=0.054$) due to considerable variation. All data represent mean ± SEM. +++$p<0.001$ overall genotype difference; *$p<0.05$, **$p<0.01$ versus corresponding WT; $^{\#\#\#}p<0.001$, versus baseline within genotype; calculated with a two-way ANOVA followed by Fisher's LSD test. Two-way ANOVA were performed for each sex, given that sexual differences in corticosterone are a well-known fact. For the S24 insert, differences were tested using unpaired t-test.

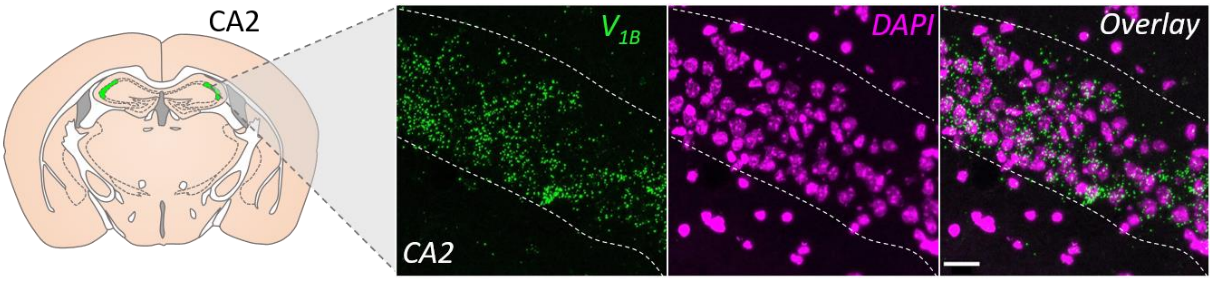

Supplementary Figure 4: Fluorescent *in situ* hybridization from female WT mouse hippocampus shows a robust and specific expression of V_{1B} (green) in the CA2, but not other areas. The section was counterstained with DAPI (magenta). Scale bar: 30µm.

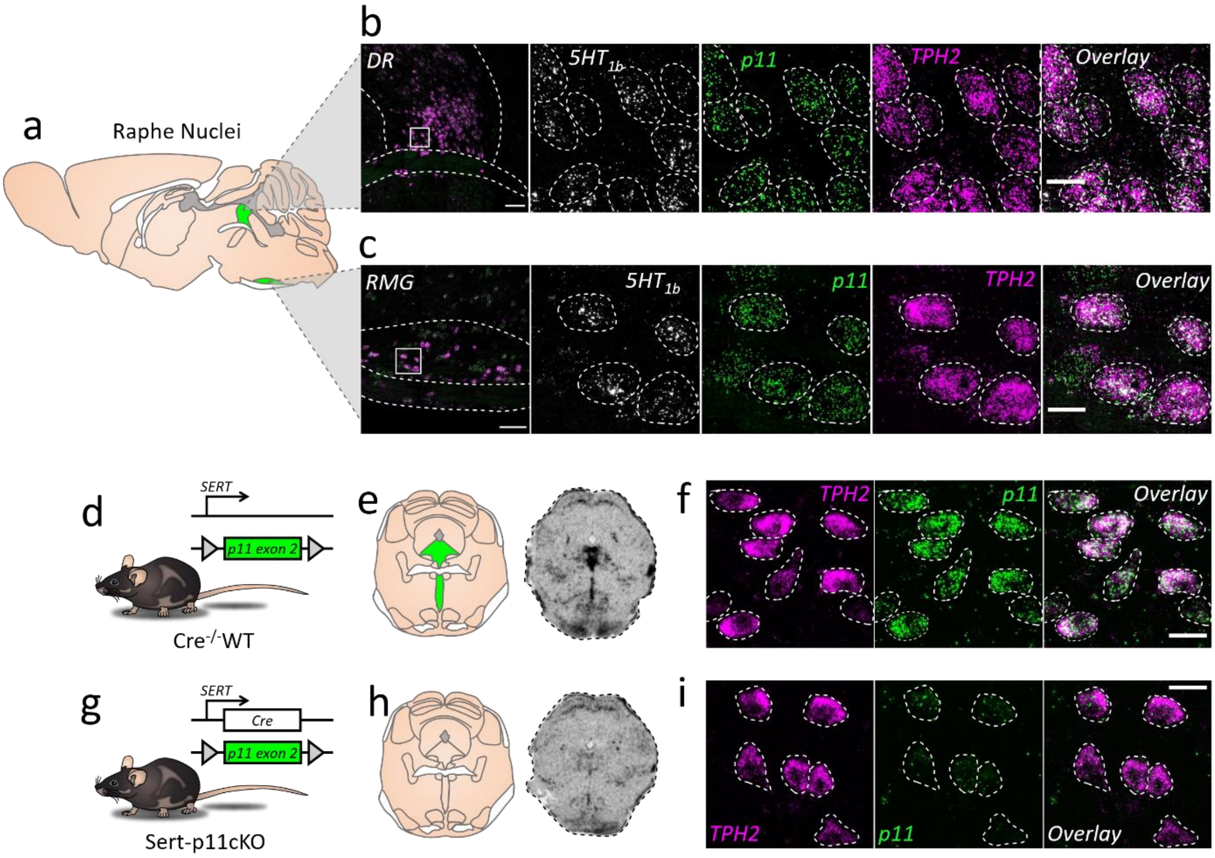

Supplementary Figure 5:

(a-c) Triple fluorescent *in situ* hybridization in a sagital section from WT mouse showing 5-HT_{1B} (white), p11 (green) and tryptophan hydroxylase (TPH2, magenta) transcripts in the dorsal raphe (**b**) and the raphe magnus nucleus (RMG) (**c**). Scale bars (**b, c**): 100 µm (on the left), 15 µm (on the right). Dashed line: 5-HT_{1B}, p11 and TPH2 co-expressing cells. **(d-i)** Selective p11-deletion in serotonergic neurons. **(d, g)** Schematic representation the genetic constructs of $Cre^{-/-}$ (WT) and SERT-p11cKO mice. **(e, h)** Diagram and representative radioactive *in situ* hybridization autoradiograms at -4.6 mm from Bregma show p11 expression in $Cre^{-/-}$ (**e**), and the selective deletion of p11 mRNA in serotonergic cell groups (**h**). The serotonergic group visible in this coronal section are: dorsal raphe (DR), median raphe (MR) and B9. **(f, i)** Double fluorescent *in situ* hybridization images showing p11 (green) and TPH2 (magenta) transcripts in dorsal raphe (DR) of $Cre^{-/-}$ (WT) (**f**) and SERT-p11cKO (**i**) mice. Scale bars: 15 µm. Dashed line: TPH2 expressing cells.

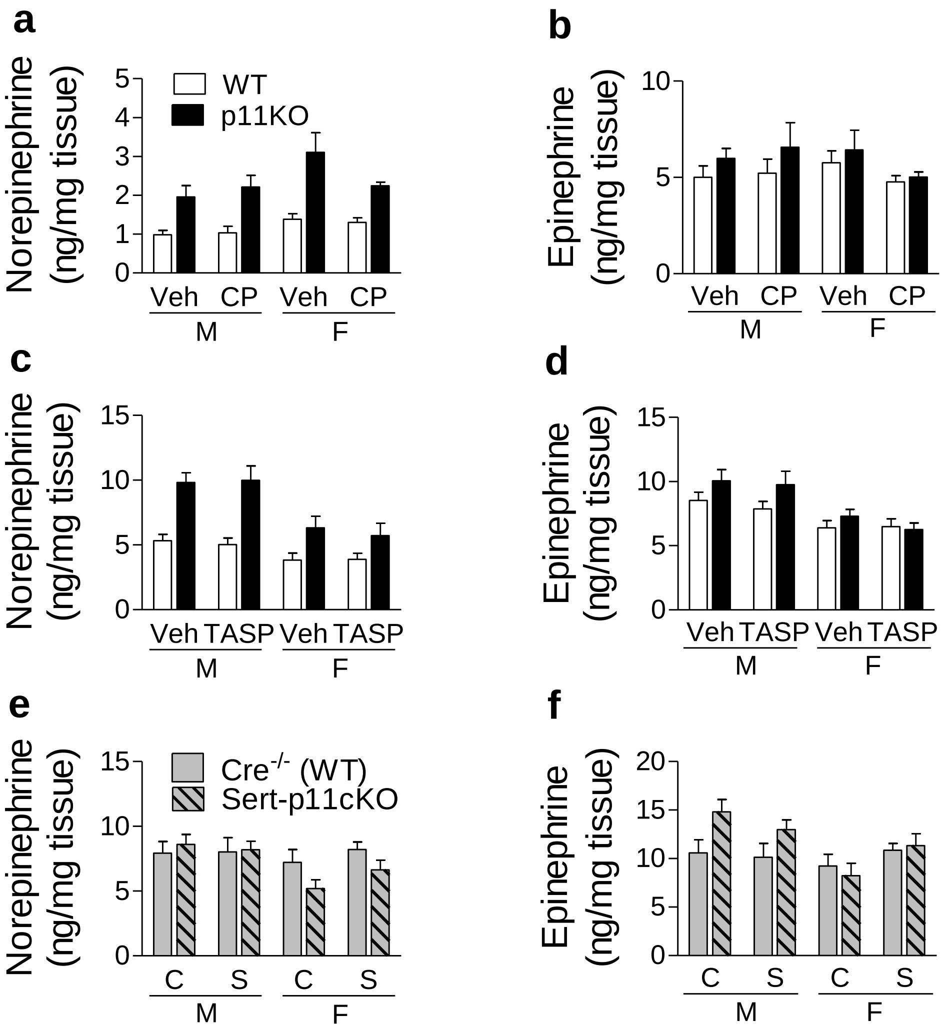

Supplementary Figure 6. Norepinephrine and epinephrine levels presented as ng/mg adrenal tissue. **(a-d)** 1 min post-stress levels of adrenal norepinephrine **(a, c)** and epinephrine **(b, d)**, measured 30 min following injection of **(a-d)** vehicle (Veh), **(a, b)** CRH1 receptor antagonist CP 154,526 (CP, 30 mg/kg, i.p.) or **(c, d)** V_{1B} antagonist, TASP 0390325 (TASP, 3 mg/kg, i.p.) in male (M) and female (F) wildtype (WT) or p11 KO mice. **(e, f)** Levels of adrenal **(e)** norepinephrine and **(f)** epinephrine, at baseline (C) or 1 min post-stress (S), in male (M) and female (F) SERTp11cKO and corresponding Cre-/- (WT) mice. Data from the same experiments are presented as percent of WT vehicle or control in Fig 3. Fig 3 also indicate relevant statistical analyses.

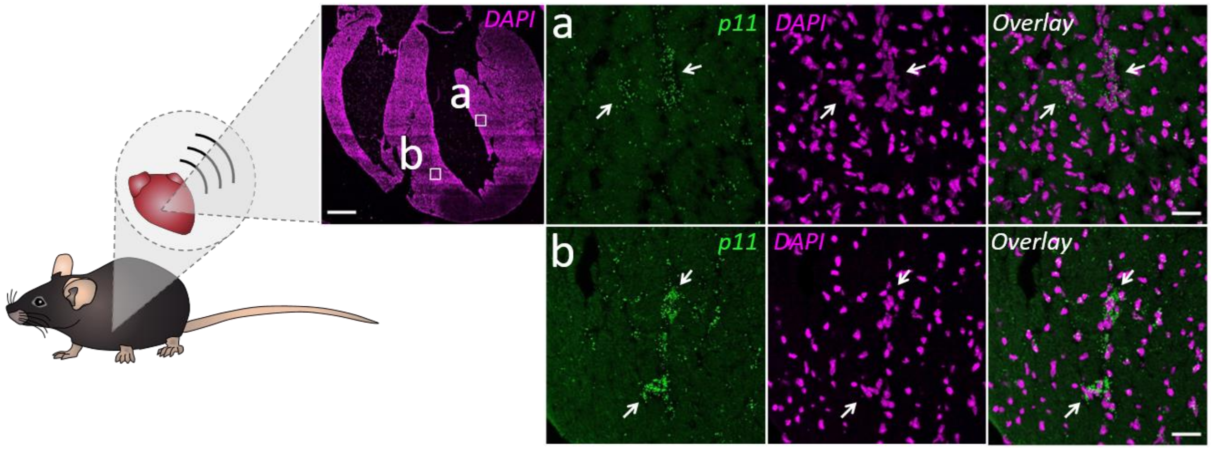

Supplementary Figure 7. Low p11 expression in murine cardiac tissue.

Fluorescent in situ hybridization of p11 mRNA (green), counterstained with DAPI (magenta).

WT mice show low levels of p11 in cardiac tissue. Scale bars: 1 mm (left), 30 μm (right).
